# Long term administration of selective NMDA GluN2B receptor blocker Ro25-6981 attenuates neurodegeneration in mouse model of spinocerebellar ataxia type 1 (SCA1)

**DOI:** 10.1038/s41420-026-03120-z

**Published:** 2026-04-13

**Authors:** Olga S. Belozor, Alexandra G. Mileiko, Lyudmila D. Mosina, Ilya G. Mikhailov, Milana S. Mikhailova, Elena D. Khilazeva, Darius A. Ox, Egor S. Grinev, Elizaveta B. Boitsova, Andrey N. Shuvaev, Anja G. Teschemacher, Sergey Kasparov, Anton N. Shuvaev

**Affiliations:** 1 Krasnoyarsk State Medical University named after Prof. V.F. Voino-Yasenetsky, Krasnoyarsk, Russia; 2 Scientific Research Institute of Medical Problems of North - a separate division of Federal Research Center “Krasnoyarsk Science Center” of the Siberian Branch of the Russian Academy of Science, Krasnoyarsk, Russia; 3 Siberian Federal University, Krasnoyarsk, Russia; 4 Institute of Biophysics of Siberian Branch of Russian Academy of Sciences, Federal Research Center “Krasnoyarsk Science Center SB RAS”, Krasnoyarsk, Russia; 5 Sirius University of Science and Technology, Sochi, Russia; 6 Krasnoyarsk Regional Clinical Hospital, Krasnoyarsk, Russia; 7 School of Psychology and Neuroscience, University of Bristol, Bristol, UK

**Keywords:** Movement disorders, Cell death in the nervous system

## Abstract

Spinocerebellar ataxia type 1 (SCA1) is caused by a CAG expansion in the gene that encodes the protein Ataxin1. Accumulation of the mutant protein in cells leads to degeneration of the cerebellum and brainstem, resulting in ataxia and culminates in failure of the circuits controlling swallowing and breathing. The nonselective NMDA receptor blocker memantine has been proposed as a potential treatment for SCA1, as it reduces excitotoxicity and neurodegeneration in other murine models of neurodegeneration. However, side effects of memantine limit its therapeutic potential, highlighting the need for more selective treatments. We have developed an SCA1 model where a lentiviral vector (LVV) selectively expresses mutant Ataxin1 in cerebellar astrocytes, triggering neuronal death through glial dysfunction and NMDA receptor-mediated excitotoxicity. Using this model, we investigate the effects of long-term administration of Ro25-6981, a selective blocker of the GluN2B subunit of glutamate receptors, typically found on extrasynaptic NMDA receptors. Chronic administration of Ro25-6981 (0.5 mg/kg day intraperitoneally) for 4 weeks prevented deterioration of motor activity in SCA1 model mice, an effect, associated with reduced neurodegeneration and decreased reactivity of Bergmann glia in the cerebellar cortex. Moreover, short-term endocannabinoid-mediated plasticity was partially preserved. Long-term blockade of NMDA receptors with Ro25-6981 caused a compensatory upregulation of expression of GluN2B and NR2A subunits. These findings suggest that specific targeting of extrasynaptic NMDA receptors with Ro25-6981 or similar drugs might offer a viable therapeutic strategy for treatment of SCA1.

## Introduction

SCA1 is a neurodegenerative disease that primarily affects the cerebellum and brainstem. It is caused by a CAG expansion in the ATXN1 gene, which encodes the protein ataxin-1. Mutant ataxin-1 has a pathologically elongated polyglutamine chain, it misfolds and forms cytotoxic aggregates [1, 2]. The mutant accumulates not only in Purkinje cells (PC), but also in other cells, including astrocytes (Bergmann glia, BG) in the cerebellum [3]. BG, affected by mutant ATXN1 becomes reactive and loses ability to take up glutamate [4]. Nearly 90% of glutamate is cleared by transporters on astrocytic end feet [5, 6]. When transporters fail, glutamate spills over from the synaptic cleft and activates extrasynaptic NMDA receptors [7] and this seems to be one of the leading mechanisms of excitotoxicity [8, 9]. Glutamate spillover also affects extrasynaptic metabotropic glutamate receptors [10]. These receptors mediate long-lasting Ca^2+^ and Na^+^ inward currents in neurons through opening of the transient receptor potential cation channel type 3 (TRPC3), this also triggers apoptotic cascades [11]. Loss of function following PC death leads to the development of the ataxic phenotype in SCA1 [12].

SCA1 remains an incurable disease. Potential therapeutic approaches for SCA1 are based on experience obtained with other neurodegenerative disorders, such as Alzheimer’s dementia. One candidate drug is memantine, a low-affinity, voltage-dependent, noncompetitive NMDA receptor antagonist [13, 14]. Memantine has been shown to prevent cell death and improve cell morphology in several mouse models of neurodegenerative diseases [15–18]. Previously, we established a model where a toxic mutant ataxin-1 bearing 85 glutamine repeats (ATXN1[Q85]) is expressed in cerebellar BG using LVVs under an astrocyte-specific promoter [19]. Mutant ataxin-1 makes BG reactive and causes excitotoxicity, which leads to PC death [20]. We tested memantine as therapeutics in this model and found that long-term memantine administration in these mice significantly improved BG and PC morphology and reduced cell death. However, memantine impaired motor activity, this was evident in both in the SCA1 model and wild-type mice. We also showed that memantine decreases glutamate release from parallel fiber (PF) terminals [21], which is likely to affect cerebellar plasticity. Impaired coordination and dizziness are common side effects in dementia patients, receiving memantine [22]. These side effects represent a serious challenge for clinical application of memantine and are particularly unwelcome in SCA1 patients.

Theoretically, selective NMDA receptor blockers that target the extrasynaptic receptors, without disrupting normal synaptic transmission [23] could help to avoid these problems. One potential candidate is Ro25-6981, a voltage-independent but activity-dependent selective blocker of the NMDA receptor containing GluN2B subunits, which are typically found in extrasynaptic locations [24, 25].

In this study, we investigated the effects of long-term Ro25-6981 administration in our SCA1 mouse model, with the focus on motor activity, synaptic transmission between PFs and PCs (PF-PC), and synaptic plasticity.

## Results

### Long-term administration of Ro25-6981 rescues ataxic phenotypes in SCA1 model mice

**Fig. 1:**
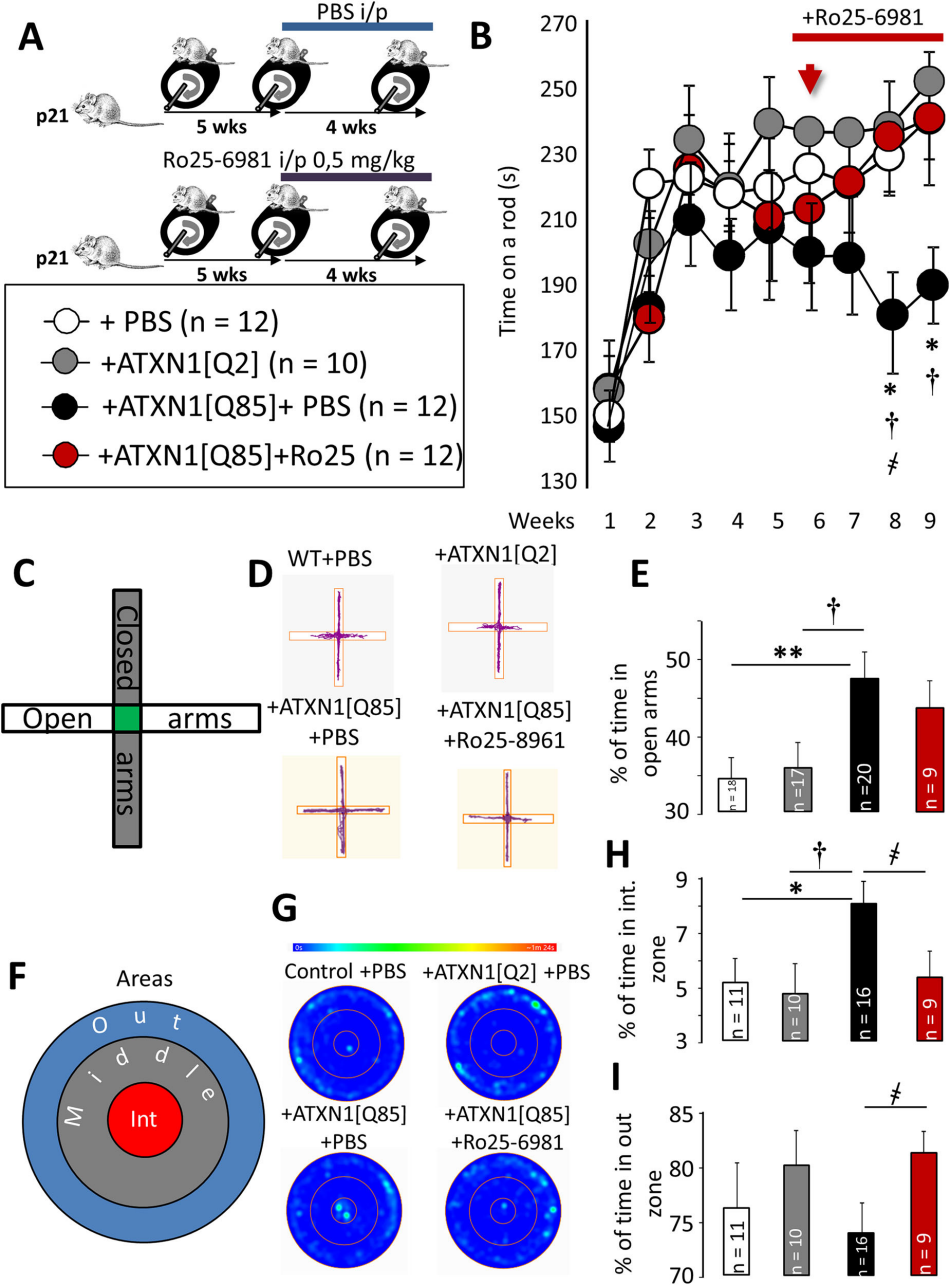
Effects of long-term Ro25-6981 administration on motor learning and anxiety in SCA1 model mice. **A** Experimental design and rotarod training protocol. **B** The diagram shows the mean ± SEM time spent on the rotarod. The arrow indicates the start of daily intraperitoneal Ro25-6981 administration. Ro25-6981 rescued the ataxic phenotype beginning from week 8 of training (P_ATXN1[Q2]/ATXN1[Q85]_ = 0.028; P_ATXN1[Q85]+PBS/ATXN1[Q85]+Ro25-6981_ = 0.035; one-way ANOVA with Tukey HSD post hoc). **C** Diagram of the elevated plus maze. **D** Cumulative movement maps of mice in the elevated plus maze arms. **E** Graph showing the mean % of time spent in the open arms. SCA1 model mice spent significantly more time in the open arms (P_ATXN1[Q2]/ATXN1[Q85]_ = 0.021; P_ATXN1[Q85]+PBS/ATXN1[Q85]+Ro25-6981_ = 0.90; one-way ANOVA with Tukey HSD post hoc). **F** Zone layout in the open field test. **G** Heat maps of mouse movement in the open field. The color scale indicates time spent in a location, ranging from blue (0 s) to red (90 s). **H** Graph showing the mean ± SEM % of time spent in the internal zone of the open field test. SCA1 model mice spent more time in the internal zone; SCA1 model mice spent less time in the external zone; Ro25-6981 returned these values to levels observed in the control groups (P_ATXN1[Q2]/ATXN1[Q85]_ = 0.042; P_ATXN1[Q85]+PBS/ATXN1[Q85]+Ro25-6981_ = 0.046; one-way ANOVA with Tukey HSD post hoc). **I** Graph showing the M ± SEM % of time spent in the external zone of the open field test. Ro25-6981 significantly increase these values (P_ATXN1[Q2]/ATXN1[Q85]_ = 0.157; P_ATXN1[Q85]+PBS/ATXN1[Q85]+Ro25-6981_ = 0.038; one-way ANOVA with Tukey HSD post hoc). *n* = number of animals tested. Statistical significance was determined by ANOVA. * – significant difference between PBS and ATXN1[Q85]-expressing mice. † – significant difference between ATXN1[Q2]-expressing and untreated ATXN1[Q85]-expressing mice. ҂ – significant difference between untreated ATXN1[Q85] mice and ATXN1[Q85] mice treated with long-term Ro25-6981. *, †, ҂*p* < 0.05; **p < 0.01.

We used our previously established SCA1 mouse model where the mutant ataxin 1 is expressed in cerebellar BG via LVVs containing astrocyte-specific GFAP promoter driving expression of ATXN1[Q85]-Flag construct [19]. LVVs were injected intracortically into molecular layer (ML) of cerebellar lobe VI of P21 mice and vial lateral diffusion spread across a significant area of cerebellar cortex (see below). This led to astrocyte-mediated cerebellar glia- and neurodegeneration, glutamate spillover–related excitotoxicity and ataxic phenotype, characteristic of SCA1. Beginning from week 5 and until week 9, some of these mice received daily intraperitoneal injections of either PBS or Ro25-6981 (0.5 mg/kg in PBS; Fig. 1A). Mice in the control group were injected with LVV GFAP-ATXN1[Q2]-Flag construct, which is not pathogenic.

LVV-induced ATXN1 expression was detected using anti-Flag staining combined with anti-GFAP staining for colocalization in the BG. The anti-Flag signal spread along the BG processes as distinctive dots. In ATXN1[Q85]-expressing mice, Flag staining was concentrated more proximally in BG processes compared to ATXN1[Q2]-expressing animals (Supplementary Fig. 1) most likely due to the aggregation of the mutant protein in the soma of the cells.

Motor learning and coordination were assessed once weekly using a rotarod test with acceleration (Fig. 1A). Nine weeks of viral transduction were sufficient to produce a prominent ataxic phenotype, similar to that observed in SCA1 knock-in (KI) model mice [26]. Normally, mice learn to stay on the rod, after ~2–3 trials/weeks their performance stabilizes and only marginally changes between weeks 4 and 9 (Supplementary Fig. 2). ATXN1[Q85]-expressing mice were not different to other groups for the first 3–4 weeks but afterwards exhibited a gradual impairment in motor coordination, which became highly significant by the eighth week, likely reflecting gradual destruction of the cerebellum (see below). Long-term administration of Ro25-6981 preserved motor performance in ATXN1[Q85]-expressing mice, at levels, comparable to those of the control group (Fig. 1B, Supplementary Fig. 2).

Repetitive rota-rod paradigm may be influenced by several aspects of behavior, in addition to motor coordination and/or balance. For this reason we also evaluated the effects of ATXN1[Q85] expression and Ro25-6981 on general motor activity of the animals and their level of anxiety.

To investigate the anxiety level, we used the elevated plus maze (EPM) test (Fig. 1C). These tests were run once during the last week of rotarod training (week 9). When mice are placed into an EPM apparatus they spend time between the central compartment and open or closed arms. Usually, time spent in central area is not included in the analysis. Naïve mice exhibit typical anxiety-like behavior in the EPM and prefer to spend more time in the closed arms (Fig. 1E) [21]. In this study time spent in closed arms was not different between any of the groups. However, mice expressing ATXN1[Q85] spent significantly more time in the open arms compared to both, PBS-injected controls and ATXN1[Q2]-expressing mice. This change was not significantly prevented by Ro25-6981 in spite of the apparent trend (Fig. 1E). At the same time, ATXN1[Q85] mice moved faster and covered longer distances in EPM compared to control groups (Supplementary Fig. 3A, B). Animals seemed to move erratically, rapidly entering and exiting different arms of the apparatus (Supplementary Fig. 3C). Ro25-6981 did not normalize these parameters but, in fact, further exacerbated them. Results of EPM test (Fig. 1D, E) suggested reduced anxiety in ATXN1[Q85]-expressing mice but, given overall change in animal behavior (Supplementary Fig. 3A–C), required additional support from another test. To this end, we performed open field test in a round arena, which measures general locomotor activity and anxiety. In this paradigm the anxiety level is assessed by measuring the fraction of time spent in the center of the arena [27] (Fig. 1F). Mice expressing ATXN1[Q85] stayed longer in the central zone compared to ATXN1[Q2]-expressing mice (Fig. 1G, H) but the average speed and total travel distance in the open field did not differ between the groups (Supplementary Fig. 3D, E). Interestingly, long-term treatment of ATXN1[Q85]-expressing mice with Ro25-6981, restored normal behavior, the time spent in the central vs external zones was similar to PBS controls and ATX1[Q2]-injected mice (Fig. 1H, I). However, Ro25-6981 did not affect their general locomotor activity (Supplementary Fig. 3D, E). Thus, the drug did antagonize some, but not all changes in animal behavior induced by ATXN1[Q85]. These results support the growing body of evidence for cerebellum involvement in control of emotions [28–30].

### Acute application of Ro25-6981 does not affect fast synaptic transmission in PF PC synapses

**Fig. 2:**
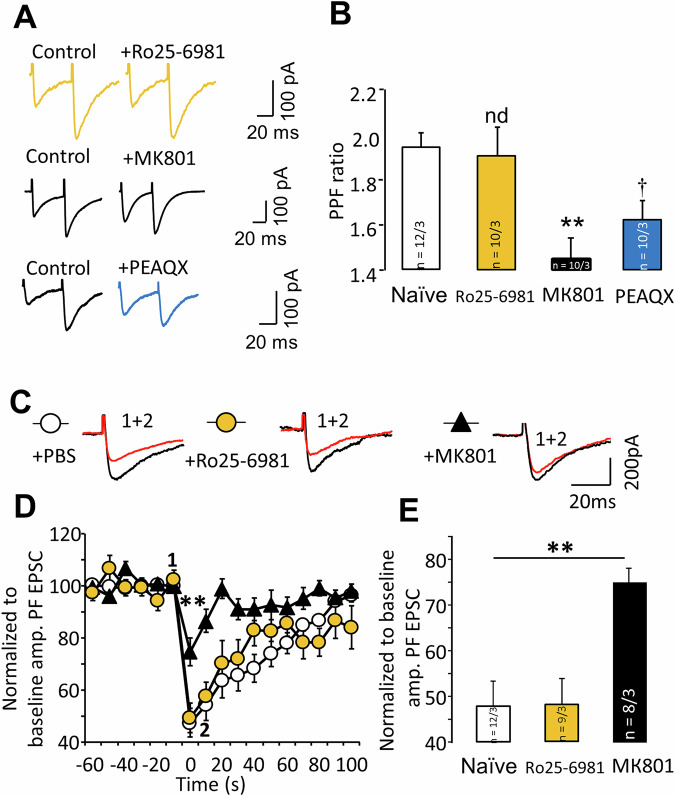
Ro25-6981, but not other NMDA blockers, preserves synaptic transmission at PF–PC synapses after acute bath application. **A** Cerebellar slices from naïve CD1 mice were used. Representative PF–EPSC traces recorded with and without NMDA blockers. **B** Bath application of MK-801 and PEAQX reduced the fast PF–EPSC PPF ratio. The graph shows mean ± SEM values of the PPF ratio (P_Ro25-6981_ = 0.90; P_MK801_ = 0.002; P_PEAQX_ = 0.046; *n* = 10; one-way ANOVA with Tukey HSD post hoc). * indicates significant differences between naïve mice and mice treated with blockers. nd – no differences; *p < 0.05, **p < 0.01. **C** Representative traces of PF–EPSCs before (1, black) and after (2, red) DSE induction. **D** Diagram of fast PF–EPSC amplitudes normalized to pre-stimulation levels (before DSE induction). **E** Graph showing the mean ± SEM of normalized fast PF–EPSC amplitudes immediately after DSE induction (point 2 in (**D**)). Representative traces of fast PF–EPSCs before (1, black) and after (1, red) DSE induction are shown in the right site. (P_Ro25-6981_ = 0.81; P_MK801_ = 0.002; one-way ANOVA with Tukey HSD post hoc). * indicates significant differences between naïve mice and mice treated with blockers. **p < 0.01. *n* = number of examined cells/animals.

Plasticity of fast EPSCs is strongly influenced by pre- and postsynaptic NMDA receptors [31]. One such phenomenon is paired pulse facilitation, whereby the EPSC, evoked by the second stimulus delivered to presynaptic terminals (PF) is greater than the first one (Fig. 2A, B). Application of the selective GluN2B blocker Ro25-6981 did not significantly affect EPSC amplitude, whereas blockade of NR2A-containing NMDA receptors with a selective antagonist 0.3 μM PEAQX or the non-selective blocker 10 μM MK801 significantly suppressed PF-evoked EPSCs (Supplementary Table 1). Moreover, both MK-801 and PEAQX strongly reduced the PPF ratio (Fig. 2A, B). Thus, blockade of GluN2B receptor blockers tested, only Ro25-6981 spares this form of synaptic plasticity in cerebellum, in contrast to non-selective blockers, like MK801.

Brief depolarization of PC (0 mV for 5 s) triggers Ca^2+^ dependent release of endocannabinoids from the postsynaptic neuron. Endocannabinoids then act retrogradely to suppress glutamate release from PF terminals, producing short-term presynaptic depression. Bath application of 0.5 μM Ro25-6981 had no discernible effect on it. By contrast, the broad-spectrum NMDA-receptor antagonist MK-801 (10 μM) impaired this type of presynaptic regulation: after depolarization, fast PF-EPSC amplitude fell to roughly three-quarters of baseline (Fig. 2C–E) [21]. Thus, here again, Ro25-6981 seems to spare normal physiological mechanisms operating in these synapses.

**Fig. 3:**
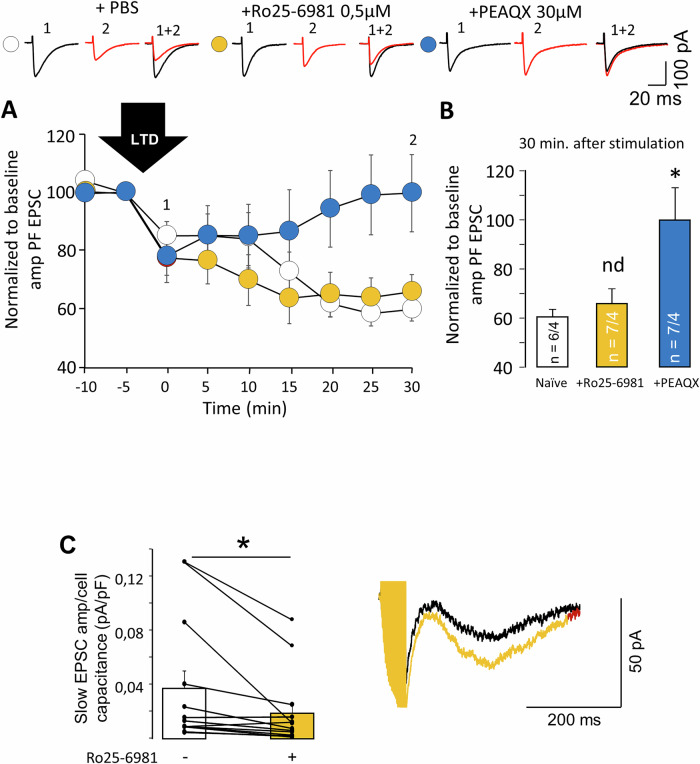
Acute application of Ro25-6981 does not affect long-term synaptic plasticity, but decrease of S-EPSCs. **A** Diagram of normalized fast PF–EPSC amplitudes before and 30 min after LTD induction from naïve PCs and after bath application of PEAQX or Ro25-6981. **B** The graph on the right shows normalized mean ± SEM amplitude 30 min after LTD induction (P_PEAQX_ = 0.019; P_Ro25-6981_ = 0.89; two-way ANOVA with Tukey HSD post hoc). *n* = number of examined cells/animals. Representative traces before (1, black) and 30 min after (2, red) LTD induction are shown above the diagrams. **C** To assess glutamate spillover and its uptake by BG, we applied bursts of 25 stimuli to PFs. The averaged and individual S-EPSCs amplitudes of the response to the burst before and after Ro25-6981 application. S-EPSC amplitude was normalized to cell capacitance to account for the cell size (left). Representative current traces are shown on the right (*P* = 0.029; paired Student’s *t* test). *p < 0.05.

Yet another typical feature of PF-PC synapses is long-term depression (LTD), which can be evoked by delivering 30 stimuli at 1 Hz to PF while depolarizing the PC to +20 mV [32]. Bath application of nonselective NMDA blockers such as memantine or APV blocks this LTD, indicating involvement of NMDA receptors in its induction [21, 33]. In the presence of 0.3 μM PEAQX, LTD protocol could not be induced while Ro25-6981 did not prevent LTD induction (Fig. 3A, B and Supplementary Fig. 4).

When glutamate spreads from the synapse onto adjacent neuronal membranes, it can engage relatively slow, but powerful cationic currents. At least two classes of glutamate receptors are involved in these currents. In addition to extrasynaptic GluN2B-containing NMDA receptors, extrasynaptic metabotropic mGluR1 activate of TRPC3-mediated currents. These conductances shape the “slow excitatory postsynaptic currents” (S-EPSCs) sensitive to blockers of extrasynaptic mGluR1 and NMDA receptors [21, 34]. These currents, which load cells with Na^+^ and Ca^2+^ could be one of the key factors, contributing to excitotoxicity and Purkinje cell (PC) death. We used brief PF tetanic stimulation to induce glutamate spillover and activate extrasynaptic receptors [26]. Currents were normalized to cell capacitance to account for the size of each cell. Ro-6981 (0.5 μM) strongly reduced amplitude of S-EPSCs (Fig. 3C).

These results demonstrate that Ro25-6981 can reduce consequences of glutamate spillover by targeting extrasynaptic GluN2B-containing NMDA receptors but does not interfere with synaptic transmission and plasticity in PF-PC synapses.

### Long-term Ro25-6981 administration decreases reactive gliosis and neurodegeneration in cerebellum of SCA1 model mice

**Fig. 4:**
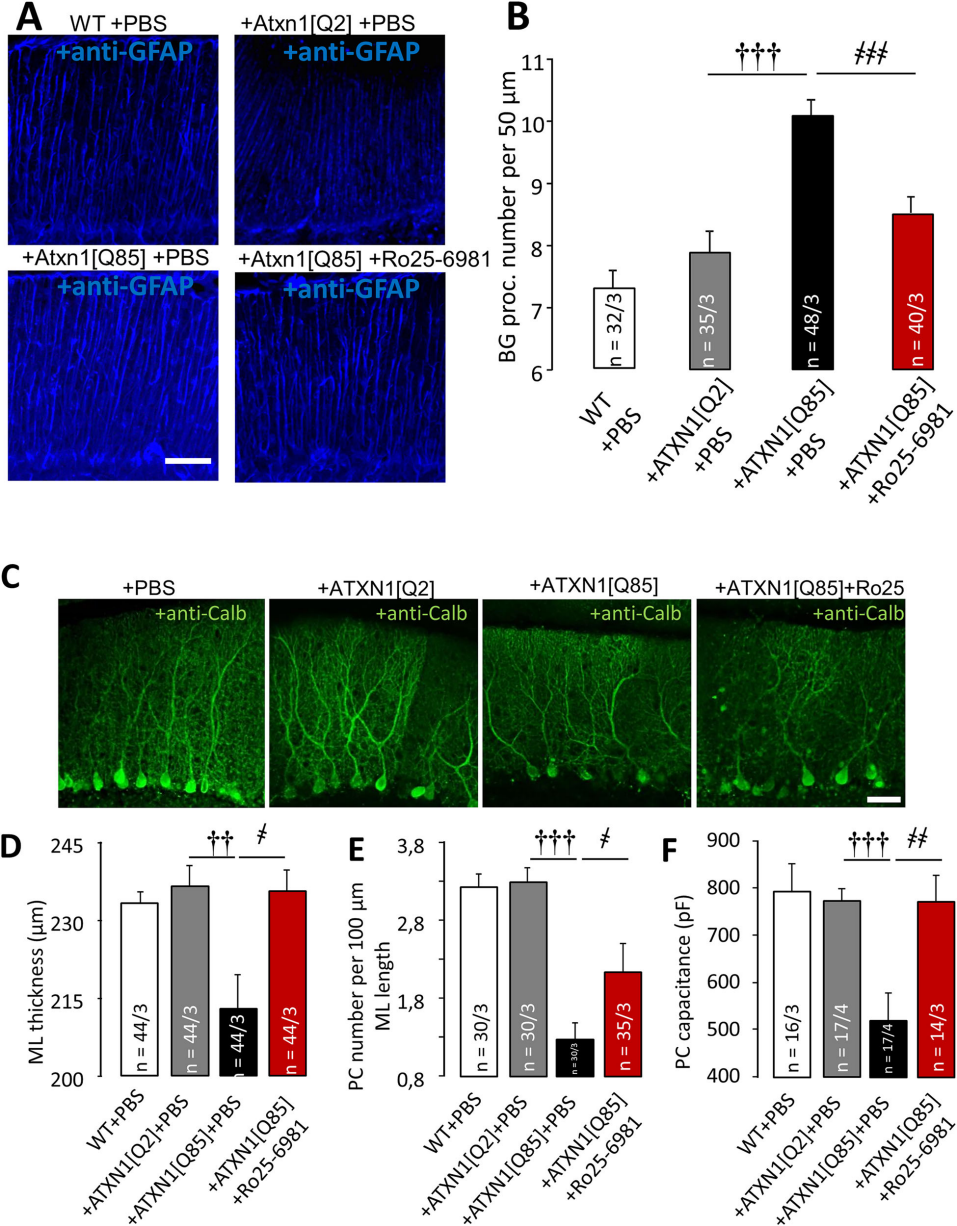
Long-term Ro25-6981 administration reduces neurodegeneration in the cerebellar cortex of ATXN1[Q85]-expressing mice. **A** Anti-GFAP staining reveals reactive BG morphology in SCA1 model mice. Representative images are shown. Scale bar: 50 μm. **B** Mean ± SEM number of BG processes per 50 μm of length (P_ATXN1[Q2]/ATXN1[Q85]_ = 1.83 ×10⁻⁶; P_ATXN1[Q85]+PBS/ATXN1[Q85]+Ro25-6981_ = 7.58 × 10⁻⁵; one-way ANOVA with Tukey HSD post hoc). **C** Anti-calbindin staining visualizes PC morphology. Scale bar: 50 μm. **D** Graph showing the mean ± SEM thickness of the ML in μm (P_ATXN1[Q2]/ATXN1[Q85]_ = 0.009; P_ATXN1[Q85]+PBS/ATXN1[Q85]+Ro25-6981_ = 0.023; one-way ANOVA with Tukey HSD post hoc). **E** Graph showing the mean ± SEM number of PCs per 100 μm (P_ATXN1[Q2]/ATXN1[Q85]_ = 2.7 ×10⁻⁶; P_ATXN1[Q85]+PBS/ATXN1[Q85]+Ro25-6981_ = 0.046; one-way ANOVA with Tukey HSD post hoc). *n* = number of examined areas/animals. Statistical significance was determined using one-way ANOVA. * – *p* < 0.05 between ATXN1[Q2]- and ATXN1[Q85]-expressing mice. **F** PC capacitances in various groups (P_ATXN1[Q2]/ATXN1[Q85]_ = 0.0004; P_ATXN1[Q85]+PBS/ATXN1[Q85]+Ro25-6981_ = 0.0043; one-way ANOVA with TukeyHSD post hoc). † – significant difference between ATXN1[Q2]-expressing and untreated ATXN1[Q85]-expressing mice. ҂ – significant difference between untreated ATXN1[Q85] mice and ATXN1[Q85] mice treated with long-term Ro25-6981. ҂*p* < 0.05, ҂҂, ††*p* < 0.01, and ҂҂҂, †††*p* < 0.001.

Preservation of rota-rod performance in our SCA1 model mice after Ro25-6981 administration may reflect improvements in BG and PC survival in the presence of the pathological allele. GFAP staining was used to visualize BG and assess their morphology (Fig. 4A). In ATXN1[Q85]-expressing mice, BG were reactive: the number of BG processes per 100 µm increased. Long-term administration of Ro25-6981 decreased the number of BG processes (Fig. 4B).

Calbindin staining was used to visualize PCs. Expression of ATXN1[Q85] in BG resulted in neurodegeneration, evidenced by reduced the thickness of ML (Fig. 4C, D). PC number and capacitance were also reduced in these animals (Fig. 4E, F). Ro25-6981 significantly improved PC morphology and reduced neurodegeneration, leading to an increase in ML thickness (Fig. 4D), increased PC number (Fig. 4E) and capacitance, which is a measure of the surface area of their membranes (Fig. 4F).

### Long-term Ro25-6981 administration prevents deterioration of synaptic plasticity in PF-PC synapses in ATXN1[Q85]-expressing mice

Expression of mutant Ataxin-1 in BG did not significantly compromise fast transmission in synapses between PF and PC (Supplementary Table 2 and Supplementary Fig. 5). Although the average amplitude of EPSC appeared reduced in ATNX1[Q85] – injected mice, these changes did not reach statistically significant level, irrespective of the statistical tests chosen (ANOVA or t-tests between individual groups), even though each group included at least 14 experiments. Therefore, we interpret further results based on the assumption, that in these mice fast EPSCs, mediated largely by AMPA receptor currents are largely preserved.

**Fig. 5:**
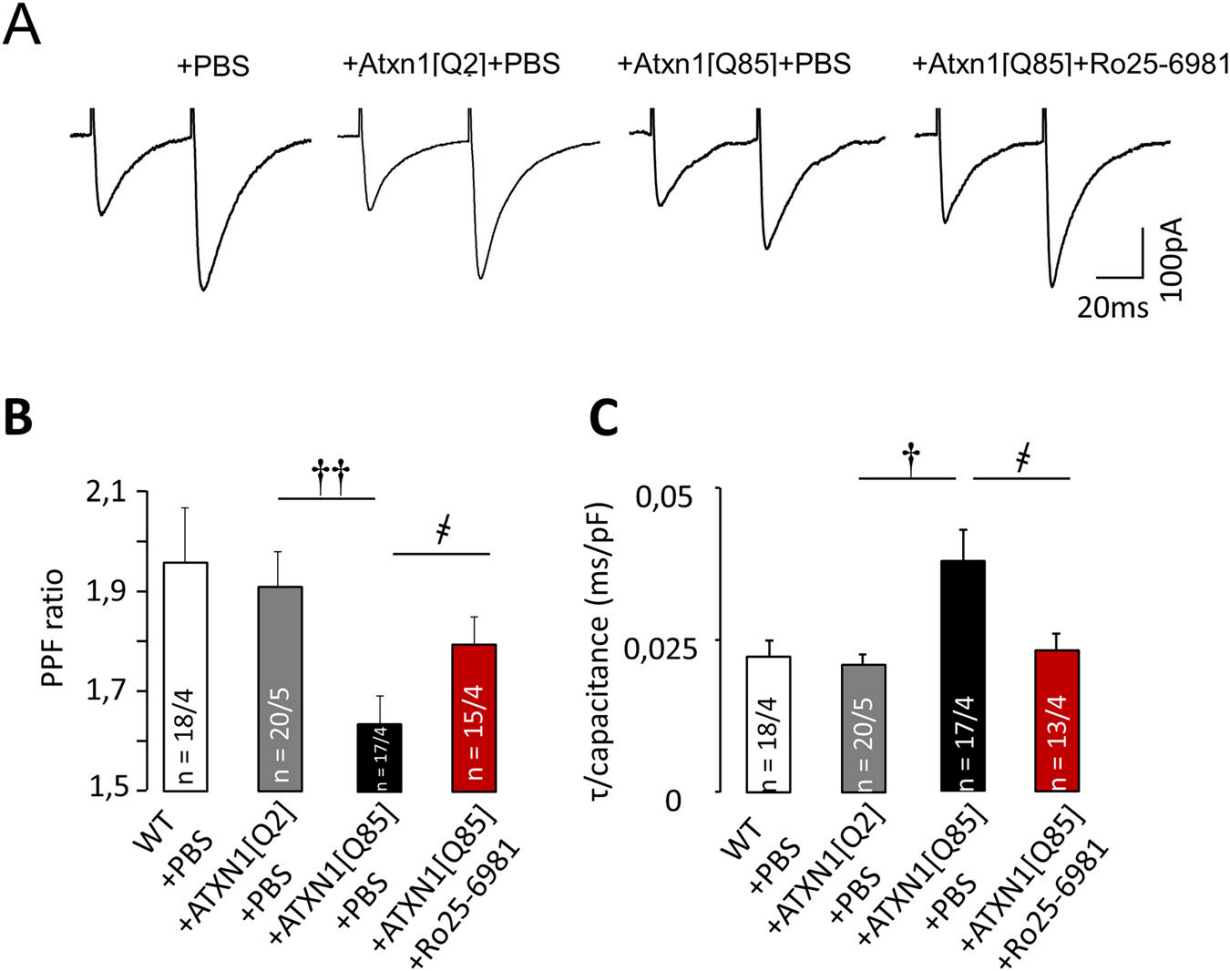
Chronic administration of Ro25-6981 prevents deterioration of PPF, caused by expression of ANXN1[Q85]. **A** Representative traces of fast PF–EPSCs recorded from PCs of SCA1 model mice. **B** PPF ratio in SCA1 model mice, with and without Ro25-6981 administration (P_ATXN1[Q2]/ATXN1[Q85]_ = 0.029; P_ATXN1[Q85]+PBS/ATXN1[Q85]+Ro25-6981_ = 0.046; one-way ANOVA with Tukey HSD post hoc). **C** Decay time normalized to cell capacitance (P_ATXN1[Q2]/ATXN1[Q85]_ = 0.018; P_ATXN1[Q85]+PBS/ATXN1[Q85]+Ro25-6981_ = 0.039; one-way ANOVA with Tukey HSD post hoc). *n* = number of examined cells/animals. One-way ANOVA used to test for significant differences. † – significant difference between ATXN1[Q2]-expressing and untreated ATXN1[Q85]-expressing mice. ҂ – significant difference between untreated ATXN1[Q85] mice and ATXN1[Q85] mice treated with long-term Ro25-6981. ҂ and †*p* < 0.05; ††*p* < 0.01.

We performed several tests to assess synaptic plasticity at PF–PC synapses. PPF, induced by paired stimulation was significantly decreased in the ATXN1[Q85]-expressing group in comparison to ATXN1[Q2]-expressing mice and animals treated with PBS. Treatment with Ro25-6981 significantly prevented this deterioration, although did not completely rescue PPF ratio (Fig. 5A, B). Similarly, a dramatic increase in decay time of first EPSC normalized to cell capacitance (τ/capacitance) in ATXN1[Q85]-expressing mice was prevented by Ro25-6981 treatment (Fig. 5C).

**Fig. 6:**
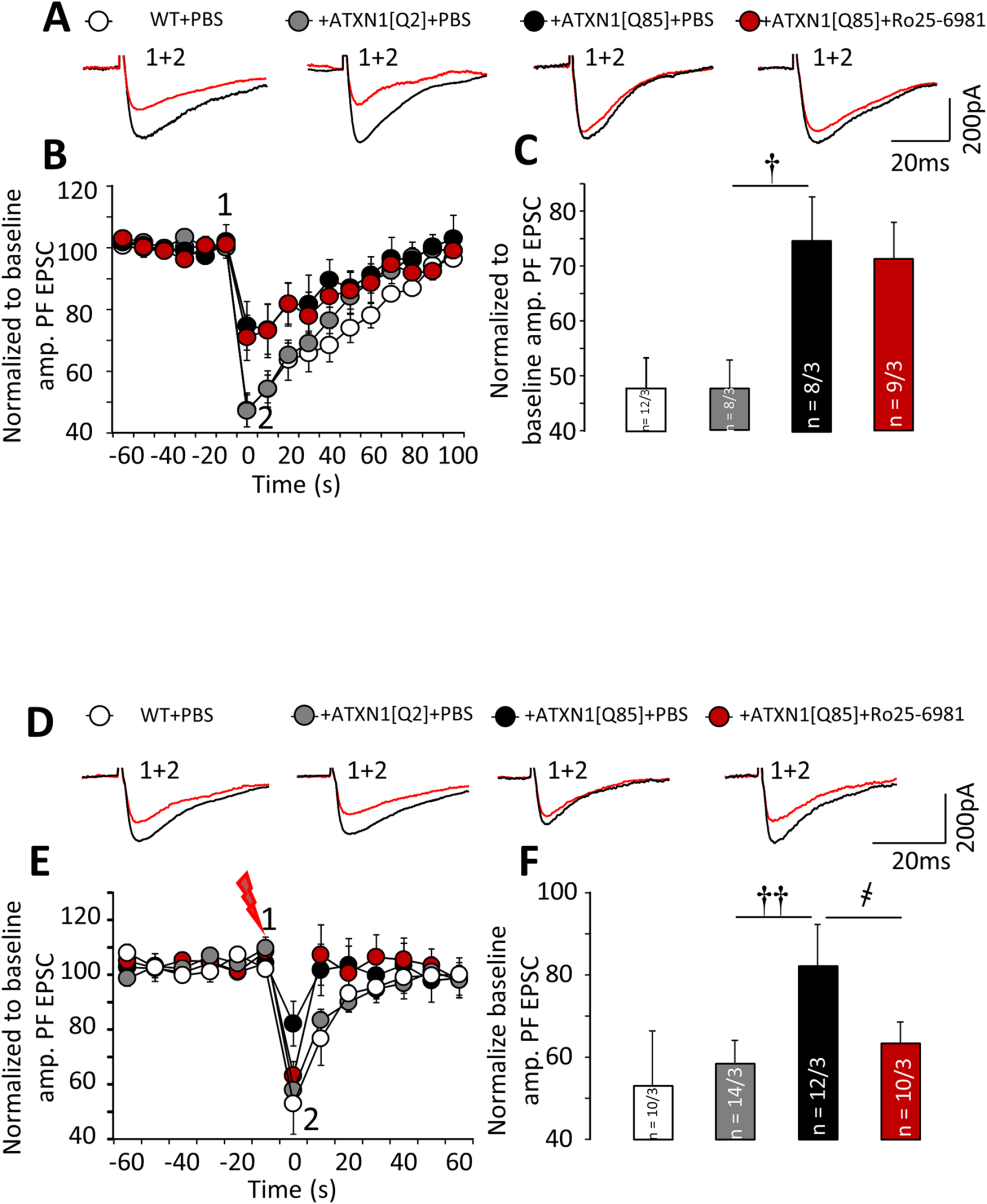
Long-term Ro25-6981 administration partially preserves endocannabinoid-mediated presynaptic inhibition in ATXN1[Q85]-expressing mice. **A** Representative traces of PF–EPSCs before (1, black) and after (2, red) DSE induction. **B** Dynamics of PF–EPSC amplitudes normalized to pre-stimulation levels (before DSE induction). **C** Normalized amplitudes of PF–EPSC immediately after DSE induction (point 2 in (**E**)) (P_ATXN1[Q2]/ATXN1[Q85]_ = 0.043; P_ATXN1[Q85]+PBS/ATXN1[Q85]+Ro25-6981_ = 0.98; one-way ANOVA with TukeyHSD post hoc). *n* = number of examined cells/animals. **D** Representative traces of PF–EPSCs before (black) and after (red) induction. **E** PF–EPSC amplitudes normalized to pre-stimulation levels (before induction). **F** PF–EPSC amplitudes immediately after induction (point 2 in (**E**)) (P_ATXN1[Q2]/ATXN1[Q85]_ = 0.024; P_ATXN1[Q85]+PBS/ATXN1[Q85]+Ro25-6981_ = 0.023; one-way ANOVA with Tukey HSD post hoc)). † – significant difference between ATXN1[Q2]-expressing and untreated ATXN1[Q85]-expressing mice. ҂ – significant difference between untreated ATXN1[Q85] mice and ATXN1[Q85] mice treated with long-term Ro25-6981. †*p* < 0.05, ҂҂, ††*p* < 0.01.

Release of endocannabinoids and retrograde presynaptic inhibition can be induced by applying a depolarizing current to a patched PC (Fig. 2C–E and Fig. 6A–C) or brief tetanic stimulation of PF (Fig. 6D–F) [35]. In either case, EPSC are reduced for a few seconds and then recover. Both varieties of endocannabinoid-induced presynaptic inhibition were significantly compromised in mice, expressing pathological ATXN1. Administration of Ro25-6981 did not prevent reduction in depolarization-induced presynaptic inhibition but completely rescued inhibition, induced by afferent stimulation (Fig. 6). It could be argued that of these two protocols, inhibition, induced by depolarization is less informative, since the cell has to be held at 0 mV for 5 s, which overloads PC with Ca^2+^, which is clearly non-physiological.

**Fig. 7:**
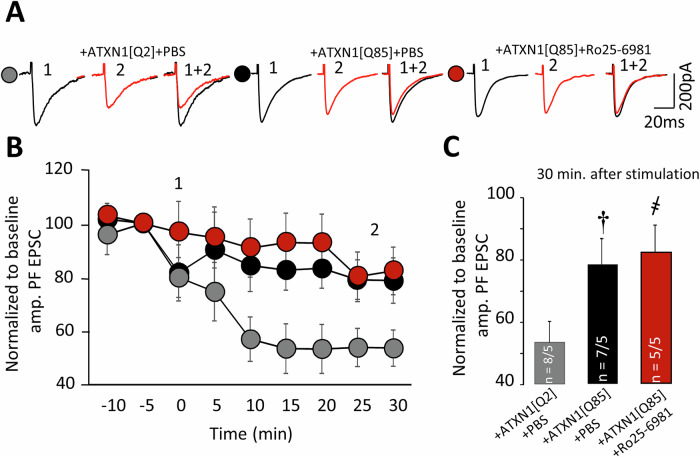
Long-term Ro25-6981 administration does not prevent LTD failure in ATXN1[Q85]-expressing mice. **A** Representative traces before (1, black) and 30 min after (2, red) LTD induction. Normalized PF–EPSC amplitudes before and 30 min after LTD induction from naïve PCs and after bath application of PEAQX or Ro25-6981. **B** Diagram of normalized fast PF–EPSC amplitudes before and 30 min after LTD induction in PCs of SCA1 model mice. **C** Graph showing the mean ± SEM of normalized fast PF–EPSC amplitudes 30 min after LTD induction (P_ATXN1[Q2]/ATXN1[Q85]_ = 0.003; P_ATXN1[Q85]+PBS/ATXN1[Q85]+Ro25-6981_ = 0.95; one-way ANOVA with Tukey HSD post hoc). *n* = number of examined cells/animals. Statistical significance was determined using one-way ANOVA. † – significant difference between ATXN1[Q2]-expressing and untreated ATXN1[Q85]-expressing mice. ҂ – significant difference between untreated ATXN1[Q85] mice and ATXN1[Q85] mice treated with long-term Ro25-6981. ҂ and †*p* < 0.05.

LTD, induced by pairing of rare presynaptic pulses with postsynaptic depolarization was severely compromised by expression of ATXN1[Q85] but treatment with Ro25-6981 did not prevent this deficiency (Fig. 7).

### Long-term Ro25-6981 administration prevents potentiation of S-EPSC in SCA1-model mice

**Fig. 8:**
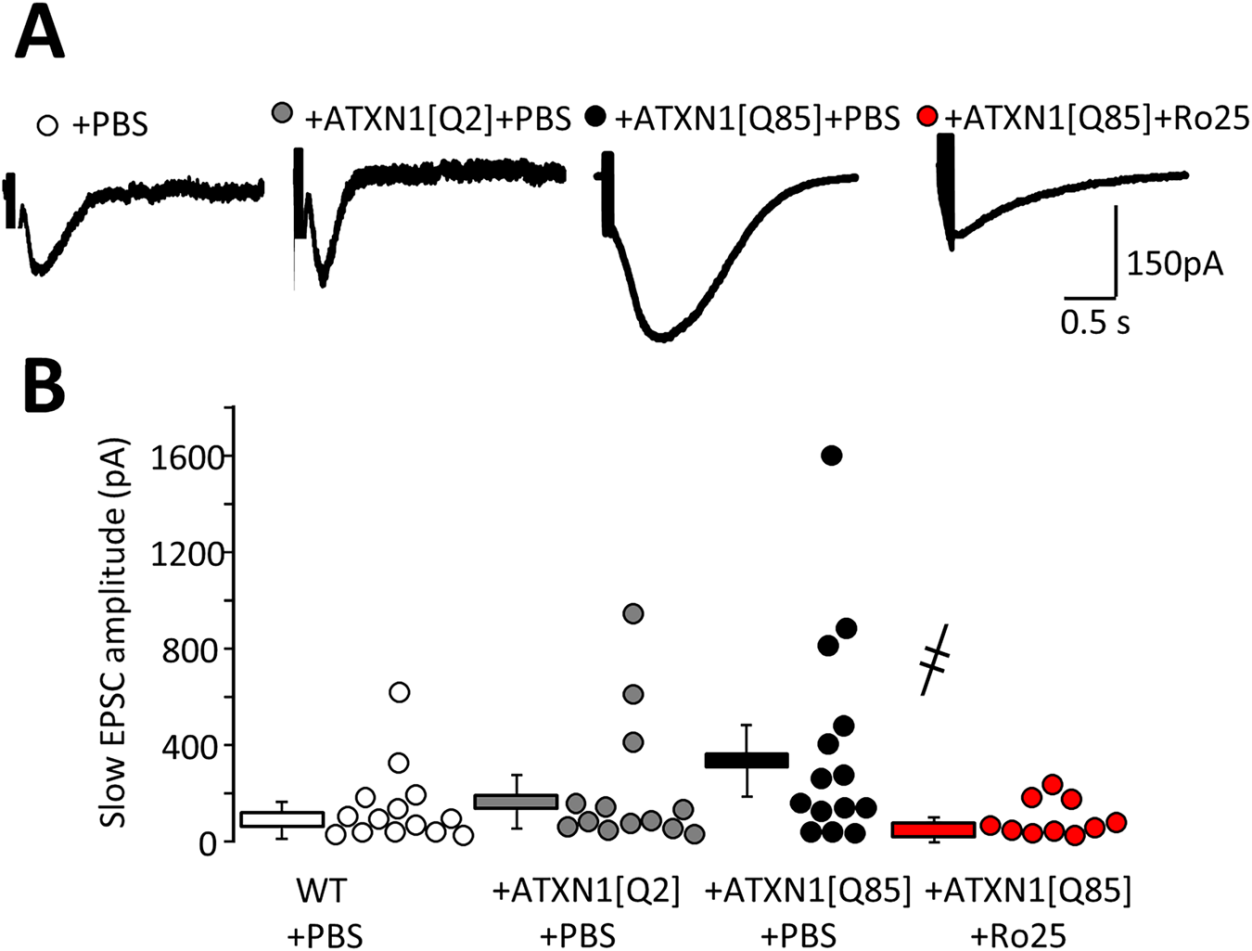
Long-term Ro25-6981 administration prevents potentiation of S-EPSCs in SCA1 model mice. **A** Representative S-EPSCs. **B** Individual S-EPSC amplitudes and averaged (mean ± SEM) of S-EPSC amplitudes are shown (P_ATXN1[Q2]/ATXN1[Q85]_ = 0.43; P_ATXN1[Q85]+PBS/ATXN1[Q85]+Ro25-6981_ = 0.03; one-way ANOVA with Tukey HSD post hoc). ҂ – significant difference between untreated ATXN1[Q85] mice and ATXN1[Q85] mice treated with long-term Ro25-6981. ҂ *p* < 0.05.

Large S-EPSC, induced by the glutamate spilled over when PF are stimulated at high frequency were greatly increased in ATXN1[Q85]-expressing mice. Even though the scatter in individual datapoints was large this increase was statistically significant and completely prevented by Ro25-6981 administration (Fig. 8A, B). No statistically significant changes in S-EPSC kinetics (decay time) were found, although with such large differences in overall amplitude this conclusion could only be made with caution (Supplementary Fig. 6).

### Changes in expression of NMDA receptor subunits and TRP3 in SCA1-model mice

S-EPSC are mediated by combined action of extrasynaptic NMDA receptors and TRPC3 currents, which are controlled by mGluR. We found that at mRNA level expression of TRPC3 was reduced in ATXN1[Q85]-expressing mice (Supplementary Fig. 7). Immunofluorescent signal from both NR2 subunits was increased in these mice, and treatment with Ro25-6981 increased it even further (Supplementary Fig. 8A–D).

## Discussion

In this study, we focused on the behavioral and cellular effects of long-term Ro25-6981 administration in a model of SCA1, in which expression of the pathological Q85 allele is specifically targeted to BG using an LVV, as described earlier [4, 23]. Previously, using this model, we found that although long-term administration of the non-selective NMDA receptor blocker memantine significantly improved BG and PC morphology and prevented cell death, but it also additionally impaired motor performance. We reasoned that block of synaptic NMDA-mediated transmission could be detrimental to cerebellar plasticity and function [21]. Therefore, selective blockers of extrasynaptic NMDA receptors, which are activated by glutamate spillover could potentially have fewer or no side effects. Ro25-6981 is a blocker of the GluN2B subunit of the NMDA receptor, which is predominantly expressed at extrasynaptic sites [36].

In our SCA1 model, ATXN1 expression in BG was observed as co-localization of anti-Flag and anti-GFAP signals in the radial processes of the molecular layer (Supplementary Fig. 1). Expression was not limited to the injected lobules 5 and 6 but spread across extensive areas within the cerebellar vermis by lateral diffusion of LVV, as noted previously [21]. Such viral transfection was sufficient to induce a prominent ataxic phenotype in ATXN1[Q85]-expressing animals by the 8th week after injection (Fig. 1A, B).

In this study, we began training the animals from the first week after LVV injection. This allowed us to assess the effect of Ro25-6981 while pathological changes were in progress, beginning from an apparently healthy phenotype (week 1). Therefore, Ro25-6981 did not interfere with the acquisition of the skill to stay on the rod during the first 3 weeks when all groups performed similarly. At later stages, when worsening cerebellum function became apparent in untreated mice (weeks 7–9), treated animals performed just as good as control groups (Fig. 1A, B).

Behavior of SCA1 model animals was tested at week 9, when their cerebellar were, obviously already severely damaged. These mice had a preference for the open arms in the EPM. This preference for open and illuminated spaces, along with exploratory behavior, is usually interpreted as evidence of reduced anxiety (Fig. 1C–H). Treatment with Ro25-6981 partially normalized behavior of mice in EPM (Fig. 1E). Increased presence in the center of the open filed (Fig. 1F) is another evidence of reduced anxiety and this change was completely obliterated by Ro25-6981 treatment (Fig. 1I). In addition to regulating motor activity, the cerebellum also plays a role in processing emotions, including anxiety-related behavior. In particular, lobule VII is involved in anxiety regulation [37]. Previous studies in transgenic mice reported different outcomes after expression of various forms of mutant Ataxin. Brain-wide expression of an allele with a very long CAG tract (154 repeats) led to increased anxiety, while overexpression of ATXN1 [Q82] in PC selectively, led to an apparently reduced anxiety [38] and this outcome is close to what we have observed. Most likely, irrespective of whether the damage to PC is direct or induced by affected glia, the impact on the anxiety is similar. Thus, our results complement and expanded our understanding the roles of PCs in this cerebellar region. Ro25-6981 clearly reduces the damage caused by ATNX1[Q85] expression and mice behave closer to the healthy controls. Effects of Ro25-6981 could also be a result of its actions on the whole brain, rather than selectively on cerebellum. Several studies have mentioned “anxiolytic” effects of Ro25-6981 in rodent tests [39, 40] and the overall result in diseased mice may be very complex.

We compared effects of Ro25-6981 with those of MK801 (non-selective NMDA receptor blocker) and PEAQX (GluN2A-selective antagonist) on several protocols traditionally used to assess cerebellar plasticity at the level of PC. It appears that Ro25-6981 spares a number of physiological mechanisms of plasticity in PF-PC synapses, such as PPF (Fig. 2) or presynaptic endocannabinoid-induced inhibition (Fig. 2C–E), in contrast with MK801 and PEAQX. Effect of PEAQX on PPF most likely indicates that PPF requires presynaptic NMDA receptors which seem to be unaffected by Ro25-6981. While there is data demonstrating presence of functional postsynaptic NMDA receptors in synapses, formed on PC by climbing fibers [41], transmission is synapses between PF and PC seems to not involve NMDA receptors and is solely mediated by AMPA-type currents. In our experiments the first EPSCs in the PPF protocol were also insensitive to MK-801 and PEAQX (Fig. 2A).

Neither did Ro25-6981 change short term depression of EPSC induced by depolarization of a PC (Fig. 2C–E). Upon depolarization and Ca^2+^ entry PC release endocannabinoids [42] which act presynaptically to reduce glutamate release in PF-PC synapses. In contrast, MK801 drastically reduced depolarization-induced presynaptic inhibition.

LTD has been implicated in cerebellar plasticity and motor learning [43]. PEAQX inhibited it almost completely, while Ro25-6981 did not affect it in any way, indicating lack of role for extrasynaptic NMDA receptors.

However, S-EPSC, induced by brief tetanic stimulation of afferents and large spillover of glutamate were clearly sensitive to Ro25-6981 (Fig. 3C). This is important, because such extrasynaptic currents are very likely to significantly contribute to degeneration of PC by overloading them with Ca^2+^ and Na^+^ [44, 45]. It may be speculated that function of glia is particularly important in this case because it limits the spread of glutamate by active uptake via glutamate transporters, abundantly expressed on astrocytes [46]. That Ro25-6981 did not completely block these S-EPSC is not surprising, since they seem to be an integral of several processes, one of which is current via TRPC3 which can be activated by extrasynaptic mGluR [47].

Thus, of all paradigms we have used, only S-EPSC turned out to be inhibited by Ro25-6981 in wild-type tissue in accordance with the hypothesis that Ro25-6981 acts on extrasynaptic GlN2B subunit-containing NMDA receptors.

Injection of LVV and expression of pathological ATXN1 induced a progressive damage in the cerebellum [4]. While we do not have morphological data on each consecutive week, from the animal behavior (Fig. 1B) it seems that during the first 2–3 weeks mice were not yet functionally compromised but from week 7 the pathology became obvious. Since we intended to mimic a clinical scenario where drugs are prescribed at the point when the disease becomes diagnosed, but not before, we started Ro25-6981 treatment at week 6 and continued until the end of experiment (week 9) (Fig. 1A). We found that chronic Ro25-6981 administration reduced BG reactivity (Fig. 2A, B), preserved PC morphology and thus, reduced neurodegeneration in our SCA1 model (Fig. 2C–E). This may explain why treated SCA1-animals exhibited by weeks 8–9 normal motor activity, comparable to the control groups (Fig. 1). We previously demonstrated that the expression of mutant ataxin-1 disrupts passive electrophysiological properties, such as the membrane capacitance of PCs ([21] and Fig. 4F). Here we show that prolonged administration of Ro25-6981 prevented loss of membrane capacitance of PCs (Fig. 4F), which is essential for proper integration of synaptic inputs.

Treatment with Ro25-6981 preserved some of the important phenomena related to cerebellar plasticity. For example, PPF was severely reduced in untreated ATXN1[Q85]-expressing mice but significantly higher in treated animals. Decay phase of EPSC (τ/capacitance) shown on Fig. 5C was greatly increased by expression of pathological allele but normal in treated mice. This probably results from an improvement in glutamate reuptake by astrocytic processes, preserved by Ro25-6981 (Fig. 4A, B).

Interestingly, of the two protocols to induce presynaptic retrograde endocannabinoid-dependent inhibition [48], only one variant was preserved by treatment with Ro25-6981. When reduction of EPSC was induced by depolarisation of the recorded PC to 0 mV, the long-lasting decrease in EPSC was greatly reduced by expression of ATXN1[Q85] and Ro25-6981 was unable to rescue it (Fig. 6A, B). However, when PC were depolarised by a brisk tetanic stimulation of afferents, presynaptic inhibition was also severely compromised in SCA1-model mice and on this occasion, Ro25-6981 treatment completely prevented the deficiency (Fig. 6D, E). It is thought that release of endocannabinoids in both protocols is a consequence of increases in intracellular Ca^2+^ in PC and consecutive release of arachidonic acid metabolites [49]. Quite possibly, functional integrity of PC in ATXN1[Q85]-expressing, Ro25-6981-treated mice is not fully preserved, after all, many PC were lost even in treated animals (Fig. 4F). In such a case, a relatively mild protocol (synaptic stimulation) might appear intact (Fig. 6D–F) while endocannabinoid release caused by loading PC with Ca^2+^ by prolonged depolarisation could not be rescued (Fig. 6A–C).

Similarly, LTD, which heavily relays on Ca^2+^ handling in PC was nearly lost in SCA1-model mice slices and treatment with Ro25-6981 was not able to prevent this. It is thought that processes similar to LTD are important for cerebellum-dependent learning [43]. Mice clearly learned to stay on the rod during weekly rota-rod tests, which was particularly clear during the first 3 weeks (Fig. 1B). At that time they were drug free and, most likely, their cerebellar were relatively intact. It is therefore not surprising that that all groups learned to approximately same level by weeks 3–4 when their LTD could also be normal. At later stages, however, treatment with Ro25-6981 did not prevent progressive motor improvement in SCA1-model mice in spite of the failing LTD, indicating that this electrophysiological paradigm cannot be equated with behavioral outcome of the drug administration.

Perhaps, the most important difference between treated and untreated animals were the S-EPSC, induced by a protocol, designed to reveal the spillover of glutamate (Fig. 8). These currents were massively enhanced in slices from ATXN1[Q85]-expressing mice and Ro25-6981 treatment completely prevented this increase. In some cases S-EPSC in treated animals appeared longer, but this was not a statistically significant difference. We hypothesise that potentiation of S-EPSC is a consequence of glia degeneration and consequent failure of glutamate uptake mechanisms. By reaching extrasynaptic NMDA and mGluR, glutamate recruits currents, which overload PC with Ca^2+^ and Na^+^. Over time this triggers metabolic stress and degeneration. Ro25-6981 attenuates S-EPSC in acute slices (Fig. 3C) because they are partially dependent on extrasynaptic GlN2B-containing NMDA receptors but the data shown on Fig. 8A, B were obtained in slices, kept in vitro for hours, where the drug could not have feasibly persisted. Therefore, the effect of Ro25-6981 shown in Fig. 8 is a consequence of the drug preserving morphological and physiological integrity of the cerebellum, but not of a direct block of GluN2B at the time of recording. Potentiation of S-EPSC is unlikely to result from increased recruitment of TRPC3, because their expression seems to be reduced in ATXN1[Q85] expressing mice (Supplementary Fig. 7), which was also prevented by treatment. Additionally, we found that chronic treatment with Ro25-6981 might affect immunoreactivity of NR2A and GluN2B subunits of NMDA receptors (Supplementary Fig. 8). This was something unexpected, we can speculate that it is a compensatory reaction to long-term presence of the antagonist and it could have also contributed to the potentiation of S-EPSC (Fig. 8).

In summary, we found that chronic administration of Ro25-6981 is a safe and efficient way to preserve cerebellum from destruction, caused by expression of a pathological form of ATXN1 in BG. Animals, treated with Ro25-6981 for weeks retained good motor coordination on the rota-rod and several plasticity-related phenomena were preserved in their brain slices by the drug. We propose that Ro25-6981 or other selective GluN2B blockers should be further evaluated as potential therapies of SCA1.

## Materials and methods

All procedures for the care and treatment of animals were carried out according to the Institutional Animal Ethics Committee of Krasnoyarsk State Medical University and Russian public standard (33215–2014) regulations and are in line with Helsinki protocol for handling experimental animals (WMA Statement on animal use in biomedical research, 2016). Protocols were approved by the Institutional Animal Ethics Committee of Krasnoyarsk State Medical University (number 2 from 2025.03.11). All efforts were made to minimize suffering and to reduce the number of animals used in this study. 12-week-old CD1 mice were used in this study. Animals were kept on a 12-h light/dark cycle with free access to food and water.

### Lentiviral vector (LVV) production and amplification

Control non-pathogenic ATXN1[Q2] (encoding human ataxin 1 with 2 CAG repeats), and pathogenic ATXN1[Q85] (with 85 uninterrupted glutamine repeats) were fused in frame with the sequence encoding the FLAG tag at their 5′ends, initially within pcDNA3.1 expression vector (Invitrogen, Carlsbad, CA, USA). Next, Flag-ATXN1[Q2], then Flag-ATXN1[Q85] constructs were transferred into the lentiviral shuttle vector pTYF, under the control of the enhanced GFAP promoter [19]. The detailed procedure for viral vector production was described previously [50]. Titres of the LVV-GFAP-Flag-ATXN1[Q2] LVV and LVV-GFAP-Flag-ATXN1[Q85] were ~7 ×10^9^ transduction units (TU)/ml. LVV were stored at −80 °C and used within 6 months.

### LVV injections

Three-week-old (P21) naïve CD1 mice were anaesthetized by intraperitoneal injection of Zoletil (50 mg/kg). The level of anesthesia was controlled by monitoring the lack of withdrawal reflex every few minutes and the anesthetic was supplemented as required. Body temperature was maintained using a warm plate during surgical manipulations. 3 μL of LVV or PBS were slowly injected into the cortex of cerebellar vermis (lobule VI) using a 10 μL Hamilton syringe. Stereotaxic coordinates relative to bregma were: AP: −2.5 mm, ML: 0 mm, DV: 2 mm. Mice were then left to recover and used for further experiments 9 weeks after the injection. For slice experiments we used lobule VI of cerebellar slices to focus on the zones with extensive viral expression [4].

### Ro25-6981 administration

1-Piperidinepropanol, α-(4-hydroxyphenyl)-β-methyl-4-(phenylmethyl)-, (αR,βS)-, (2Z)-2-butenedioate (1:1), [R-(R*,S*)]-α-(4-Hydroxyphenyl)-β-methyl-4-(phenylmethyl)-1-piperidinepropanol maleate (Ro25-6981) (Abcam, UK, cat. No AB120290 Abcam) was dissolved in PBS and injected one a day intraperitoneally at 0,5 mg/kg from 6 to 9 weeks after LVV injection. We did not notice any changes in behavior, weight or food consumption of these animals after administration Ro25-6981.

### Rotarod test

Motor coordination was assessed by rotarod test with an acceleration protocol [3 min acceleration from 0 to 30 revolutions per minute (rpm)] that included 4 trials with a 30 min inter-trial interval (Fig. 2A). This protocol was repeated once a week for 9 weeks. The rod (Rota-Rod Neurobotics, Russia) consisted of a gridded plastic rod (3 cm in diameter, 10 cm long) flanked by two large round plates (50 cm in diameter). Time spent by the mice on the rotarod was recorded with a cutoff of 300 s. The time spent on the rod was averaged across all the trials on the same day and that value was then used for the statistical analysis [51].

### Elevated plus maze

The elevated plus maze (EPM) was used to investigate anxiety [52] at 9 week after LVV injection. The maze consisted of two open arms (6 cm × 32 cm) and two closed arms (6 cm × 32 cm with 19 cm tall opaque walls) with a center area 6 cm × 6 cm and was raised 54 cm above the floor. The surrounding room was dark, and the maze was lit by the overhead lights. A mouse was placed in the center of the maze facing an open arm and allowed to explore for 5 min. During this time, the movement was detected automatically using ANY MAZE animal video analysis system. The time spent in closed arms was recorded. The maze was cleaned with 70% ethanol before each trial [53].

### Open field test

The open field test was used to investigate anxiety at 9 week after LVV injection. We used circular open field arena (620 mm diameter, 320 mm height of walls), covered with polypropylene gray sheets. The center (internal) zone of open field arena (300 mm diameter) was outlined. Each mouse was placed in the open field arena for 10 min. Overall activity in the test was measured, and the time and distance traveled in the center and peripheral zones were noted. This paradigm is based on the idea that rodents spontaneously prefer the periphery of the apparatus to activity in the central parts of the open field. After each trial, the test chambers were cleaned with a damp towel and 1% sodium hypochlorite, followed by 70% ethanol [54].

### Acute slice preparation

Parasagittal cerebellar slices (250 μm in thickness) were prepared, and whole-cell recordings were conducted as described previously [55]. Briefly, mice were deeply anesthetised by Zoletil (50 mg/kg, Virbac, France) intraperitoneally and killed by decapitation. The whole brain was quickly dissected out and put for several minutes in an ice-cold Ringer’s solution containing (in mM): 234 sucrose, 26 NaHCO_3_, 2.5 KCl, 1.25 NaH2PO4, 11 glucose, 10 MgSO_4_, and 0.5 CaCl_2_ 0.5; pH 7.4, continuously oxygenated by 95% O_2_ and 5% CO_2_. Parasagittal slices of cerebellar vermis were made using a microslicer (Thermo Scientific; Microtom CU65). The slices were maintained in an extracellular solution containing (in mM): 125 NaCl, 2.5 KCl, 2 CaCl_2_, 1 MgCl_2_, 1.25 NaH2PO_4_, 26 NaHCO_3_, 10 D-glucose, and 0.05–0.1 picrotoxin. This solution was oxygenated continuously with a mixture of 95% O_2_ and 5% CO_2_ at room temperature for 1 h before starting the electrophysiological experiments.

### Electrophysiology

For voltage clamp whole-cell recordings from Purkinje cells (PCs) intracellular solution contained (in mM): 140 Cs-gluconate, 8 KCl, 10 HEPES, 1 MgCl2, 2 MgATP, 0.4 NaGTP, 0.2 EGTA (pH 7.3 adjusted with CsOH). The slices were maintained in an extracellular solution during recording containing (in mM): 125 NaCl, 2.5 KCl, 2 CaCl2, 1 MgCl2, 1.25 NaH2PO4, 26 NaHCO3, 10 D-glucose, and 0.05–0.1 picrotoxin. To estimate acute effect of Ro25-6981 to S-EPSC in PCs we used Mg free extracellular solution. These solutions were oxygenated continuously with a mixture of 95% O2 and 5% CO_2_. Passive electrical properties of the PCs were estimated using averaged traces of ~20 current responses evoked by hyperpolarising voltage pulses (from −70 to −80 mV, 200 ms duration). Liquid junction potentials were not corrected in this study. Analysis of electrophysiological data was performed using pClamp10 (Molecular Devices), Pachmaster software (HEKA), and Clampfit 10.5 (Axon instruments).

PCs were voltage-clamped at −70 mV to record excitatory postsynaptic currents (EPSCs) in synapses of parallel fibers (PF) and PCs (PF EPSCs). Selective stimulation of PFs was confirmed by paired-pulse facilitation (PPF) of EPSC amplitudes (at a 50-ms interstimulus interval). PPF ratio was calculated as 2nd/1st PF EPSC amplitude. For the recordings of S-EPSCs, the strength of the electrical stimulation was adjusted to produce AMPA receptor-mediated fast EPSCs of ~300 pA. We subsequently applied 2,3-dioxo-6-nitro-1,2,3,4-tetrahydrobenzo[f]quinoxaline-7-sulfonamide (NBQX, 20 µM), an AMPA-type glutamate receptor antagonist to remove interference from synaptic glutamate receptors, elicited S-EPSC by applying 25 electrical stimuli to PFs at 200 Hz.

PF EPSCs were recorded every 3 s. After monitoring basal PF EPSCs for 1 min, we applied PF burst stimulation (a train of 25 stimuli at 100 Hz) to induce SSE or single depolarizing pulse (5 s from −70 to 0 mV) to induce DSE. This opens the voltage-gated Ca^2+^ channels (VGCC) and releases endocannabinoids, which presynaptically decrease glutamate release and suppress amplitude of PF EPSC [26]. Amplitudes of subsequent PF EPSCs were normalized to the mean value of 12 responses evoked before stimulation.

For analysis of long-term depression (LTD), PF EPSCs were monitored every 10 s. LTD was induced by 30 single PF stimuli paired with single 200 ms depolarizing pulses (to +20 mV) repeated at 1 Hz [56]. Averaged amplitudes of PF EPSCs over 1 min were normalized to the baseline value, which was the average of the six responses just before the induction of LTD.

### Immunohistochemistry

For immunohistochemistry, mice were terminally anaesthetized with Zoletil and perfused transcardially with a fixative containing 4% paraformaldehyde in 0.1 M phosphate buffer. The whole brain was removed and postfixed in the same fixative for 5–6 h or overnight. The cerebellar vermis was cut into 50-µm sagittal sections. The sections were treated with rabbit monoclonal anti-Calbindin D-28 k (1:500, Cloud Clone Corp., Wuhan, China), chicken polyclonal anti-GFAP (1:1000, Abcam, Cambridge, UK), rabbit polyclonal anti-Flag (1:500, Cloud Clone Corp., Wuhan, China), rabbit polyclonal anti-GluN2B (1:500, Cloud Clone Corp., Wuhan, China), rabbit polyclonal anti-GluN2A (1:500, Cloud Clone Corp., Wuhan, China). Then antibodies visualized with Alexa Fluor 647-conjugated donkey anti-chicken IgG (1:1,000, Life Technologies), Alexa Fluor 555-conjugated donkey anti-goat IgG (1:1,000, Life Technologies) or Alexa Fluor 488-conjugated donkey anti-rabbit IgG (1:1,000, Life Technologies). The antibodies were dissolved in a PBS solution containing 2% (v/v) normal donkey serum, 0.1% (v/v) Triton X-100, and 0.05% NaN3.

### Confocal microscopy and morphometric analysis

In all groups, the cerebellar lobes 6 and 7 of the vermis cerebellum were used for comparisons. Fluorescent images were obtained using FV10i Confocal Microscope (Olympus, Tokyo, Japan). Images from the same confocal plane and under the same exposure and gain were compared to assess double labeling. The thickness and length of BG processes were measured on confocal images of sagittal cerebellar slices. The thickness of BG processes was analyzed using intensity profiles of a 50 μm line drawn across the layer where each glial process appeared as a peak of glial fibrillary acidic protein (GFAP)/Alexa 647 fluorescence, as described previously [4]. The cut-off detection of the GFAP signal was set to 30% of the maximal fluorescence intensity. The number and thickness of anti-GFAP labeled BG processes were counted as number and length of fluorescence intensity peaks on the graph. The number of PCs was measured across the Purkinje cells layer (PCL) over the 100 μm stretch of the section. The approximate length of the dendrites of the Purkinje cells was estimated from the overall thickness of the molecular layer (ML), visualized using anti-Calbindin/Alexa 488 staining as in our previous work [51]. For assessment of anti-NR1 and anti-NR2A signal intensity we converted microphotographs to 32 bit black and white images and analyzed using ImageJ software. To exclude fluorescence from ML interneurons we analyzed 25 μm^2^ areas between them. Correlated total area fluorescence (CTAF) was calculated with background subtraction (the darkest area of 25 px^2^ in the area of interest). CTAF = Integrated Density – (Area of selected cell X Mean fluorescence of background readings) [57].

### Analysis of TRPC3 mRNA expression in cerebellum

Total RNA was isolated from the cerebellar vermis using the RNA-Extran kit (Syntol, Russia) according to the manufacturer’s standard protocol. Reverse transcription was performed in a total volume of 20 μL using 1 μg of total RNA and the MMLV RT kit (Evrogen, Russia) at 40 °C for 1 h. Real-time PCR was then conducted on the resulting cDNA using the 5X qPCRmix-HS kit (Evrogen, Russia) and TaqMan Gene Expression Assay primer and probe sets (Thermo Fisher Scientific, USA) specific to TRPC3. GAPDH (Mm99999915) and ACTB (Mm02619580) were used as reference genes (Thermo Fisher Scientific, USA). Amplification was performed on the LightCycler 96 System (Roche, Switzerland). The PCR cycling conditions were as follows: 95 °C for 3 min, followed by 35 cycles of 95 °C for 30 s, 60 °C for 30 s, and 72 °C for 30 s. Relative quantification was carried out using LightCycler 96 Software (Roche, Switzerland).

### Data analysis

All data were obtained from single experimental cohorts per condition. For behavioral experiments, the biological replicate was one animal. For electrophysiological and morphometric analyses, tissue was collected from independent animals (3–5 animals per group, as indicated in figure legends). Multiple technical replicates (cells or imaging fields) were obtained from each animal. For statistical testing, technical measurements were first averaged within each animal to yield one value per animal, and statistical analyses were performed using the number of animals as the unit of analysis. Experiments and analyses were not performed under blinded conditions. Data acquisition and analysis were conducted with knowledge of group allocation.

All statistical tests were two-sided. Data distribution was assessed using visual inspection of residual plots and the Shapiro–Wilk normality test. Homogeneity of variances was evaluated using Levene’s test where appropriate. When assumptions for parametric testing were satisfied, comparisons among three or more groups were performed using one-way analysis of variance (ANOVA). When ANOVA revealed a significant main effect, Tukey’s Honestly Significant Difference (HSD) post hoc test was applied to adjust for multiple comparisons. For comparisons between two independent groups, an unpaired two-sided Student’s *t* test was used. For paired measurements obtained from the same cell before and after pharmacological manipulation, a paired two-sided Student’s *t* test was applied.

For experiments involving repeated measurements over time (e.g., rotarod performance across weeks), repeated-measures ANOVA was used with time as the within-subject factor and experimental group as the between-subject factor, followed by Tukey-adjusted post hoc comparisons where appropriate.

Data are presented as mean ± standard error of the mean (SEM). The center value in all bar graphs represents the arithmetic mean. For experiments with small biological sample sizes (*n* < 5 animals per group), individual data points corresponding to each biological replicate are shown. Exact *p*-values are reported in the figure legends or main text where appropriate. Differences were considered statistically significant at *p* < 0.05.

All statistical analyses were performed using R (R Foundation for Statistical Computing, Vienna, Austria).

## Supplementary information

Supplementary information

Sup. Firure 1

Sup. Figure 2

Sup. Figure 3

Sup. Figure 4

Sup. Fig. 5

Sup. Figure 6

Sup. Figure 7

Sup. Figure 8

Editable supplementary tables

## Data Availability

All data generated or analyzed during this study are included in this published article and its supplementary information and are available from the corresponding author on reasonable request.
