# Supplementary material for: Long term administration of selective NMDA GluN2B receptor blocker Ro25-6981 attenuates neurodegeneration in mouse model of spinocerebellar ataxia type 1 (SCA1)
Source: Cell Death Discov. 2026 Apr 13;12:228. doi: 10.1038/s41420-026-03120-z (PMC13184322; doi:10.1038/s41420-026-03120-z)
Supplement: Supplementary file 1 — Supplementary information [file 41420_2026_3120_MOESM1_ESM.docx]

**Supplementary Fig. 1.** Confocal images show anti-Flag (green) and anti-GFAP (blue) colocalization in the radial processes of BG in the cerebellar cortex of our SCA1 model mice after 9 weeks of LVV GFAP-ATXN1[Q2]-Flag and LVV GFAP-ATXN1[Q85]-Flag expression. Scale bar: 100 μm.

**Supplementary Fig. 2.** Individual time points spent by the animal on the rotarod. Shown are the 9-week data that were presented as average values ​​in Fig. 1B. Statistical significance was determined by ANOVA. * – significant difference between ATXN1[Q2]- and ATXN1[Q85]-expressing mice.

**Supplementary Fig. 3. A-C** Graphs showing the mean ± SEM animal speed (A), total distance (B) and number of entries to the arms (C) in elevated pluse maze. D-E Graphs showing the mean ± SEM animal speed (D) and total distance (E) in open field. Statistical significance was determined by ANOVA. * – significant difference between PBS and ATXN1[Q85]-expressing mice. † – significant difference between ATXN1[Q2]-expressing and untreated ATXN1[Q85]-expressing mice. ҂ – significant difference between untreated ATXN1[Q85] mice and ATXN1[Q85] mice treated with long-term Ro25-6981. *, †, ҂ p < 0.05. n = number of examined cells/animals.

**Supplementary Table 1. AMPA receptor-mediated fast PF–EPSCs in PCs of naïve CD1 mice after application of NMDA receptor blockers**. The table shows the kinetics of fast PF–EPSCs (10–90% rise time and decay time constant) and their amplitudes. Data are presented as mean ± SEM. n indicates the number of cells. Statistical significance was determined by one-way ANOVA. * or † indicate significant differences between naïve mice and mice treated with blockers (p < 0.05).

**Supplementary Fig. 4.** Graphs show the representative time course of changes in fast PF–EPSC amplitudes following conjunctive stimulation (LTD induction) in naïve mice, and after bath application of PEAQX and Ro25-6981.

**Supplementary Table 2. AMPA receptor-mediated fast PF–EPSC properties in PCs of ATXN1[Q85]-expressing mice after long-term Ro25-6981 administration.** The table includes kinetics of fast PF–EPSCs (10–90% rise time and decay time constant (τ)) and their amplitudes. Numbers in brackets are examined cells/animals. Statistical significance was determined using one-way ANOVA with Tukey HSD post hoc.

**Supplementary Fig. 5. AMPA receptor-mediated fast PF-EPSC amplitudes are not affected by Ro25-6981 treatment.** M ± SEM, In spite of the trends, no significant difference was found. n = number of examined cells/animals

**Supplementary Fig. 6.** Individual half-times of S-EPSC are shown with the Mean ± SEM values (P_ATXN1[Q2]/ATXN1[Q85]_ = 0.047; P_ATXN1[Q85]+PBS/ATXN1[Q85]+Ro25-6981_ = 0.95; one-way ANOVA with Tukey HSD post hoc). * – significant difference between PBS and untreated ATXN1[Q85]-expressing mice. † – significant difference between ATXN1[Q2] mice and untreated ATXN1[Q85] mice. * and † p < 0.05.

**Supplementary Fig. 7. Real Time PCR analysis of mRNA TRPC3 expression in various groups of mice.** Changes in mRNA in cerebellum vermis were measured using ACTB and GAPDH as reference housekeeping genes, M ± SEM (P_PBS/ATXN1[Q85]_ = 0.005; P_ATXN1[Q2]/ATXN1[Q85]_ = 0.037; P_ATXN1[Q85]+PBS/ATXN1[Q85]+Ro25-6981_ = 0.033). n = number of examined areas/animals. Significance was determined using one-way ANOVA with Tukey HSD post hoc. * - significant differences between PBS and ATXN1[Q85]-expressing mice. † – significant differences between ATXN1[Q2]- and ATXN1[Q85]-expressing mice. ҂ – significant differences between treated and untreated ATXN1[Q85]-expressing mice. **, p <0.01; †, ҂ p < 0.05; † p < 0.05.

**Supplementary Fig. 8 Long-term Ro25-6981 administration increases expression of NR2A and GluN2B subunits in the cerebellar cortex of ATXN1[Q85]-expressing mice.** (A) Cerebellar slices were stained with anti–GluN2B to assess changes in subunit expression in SCA1 model mice and after Ro25-6981 treatment. Representative confocal images are shown. To exclude intensive fluorescence from GluN2B positive interneurons, 20 × 20 μm² areas from the upper and lower ML were used for quantification. Scale bar: 100 μm. (B) Graphs showing the mean ± SEM of anti-GluN2B fluorescence intensity normalized to WT+PBS in the ML (P_ATXN1[Q2]/ATXN1[Q85]_ = 0.019; P_ATXN1[Q85]+PBS/ATXN1[Q85]+Ro25-6981_ = 1.5 × 10⁻¹^8^). n = number of examined areas/animals. Statistical significance was determined using one-way ANOVA with Tukey HSD post hoc. † – significant differences between ATXN1[Q2]- and ATXN1[Q85]-expressing mice. ҂ – significant differences between treated and untreated ATXN1[Q85]-expressing mice. ҂ p < 0.05; ҂҂҂, ††† p < 0.001. (C) Similar to A and B but slices were stained with anti–GluN2A antibodies. Scale bar: 100 μm (P_ATXN1[Q2]/ATXN1[Q85]_ = 0.00022; P_ATXN1[Q85]+PBS/ATXN1[Q85]+Ro25-6981_ = 5.1 × 10⁻¹^2^) (one-way ANOVA with Tukey HSD post hoc). n = number of examined areas/animals. Statistical significance was determined using one-way ANOVA. † – significant differences between ATXN1[Q2]- and ATXN1[Q85]-expressing mice. ҂ – significant differences between treated and untreated ATXN1[Q85]-expressing mice. ҂҂҂, ††† p < 0.001.
