# Supplementary figures and images for: Long term administration of selective NMDA GluN2B receptor blocker Ro25-6981 attenuates neurodegeneration in mouse model of spinocerebellar ataxia type 1 (SCA1)
Source: Cell Death Discov. 2026 Apr 13;12:228. doi: 10.1038/s41420-026-03120-z (PMC13184322; doi:10.1038/s41420-026-03120-z)

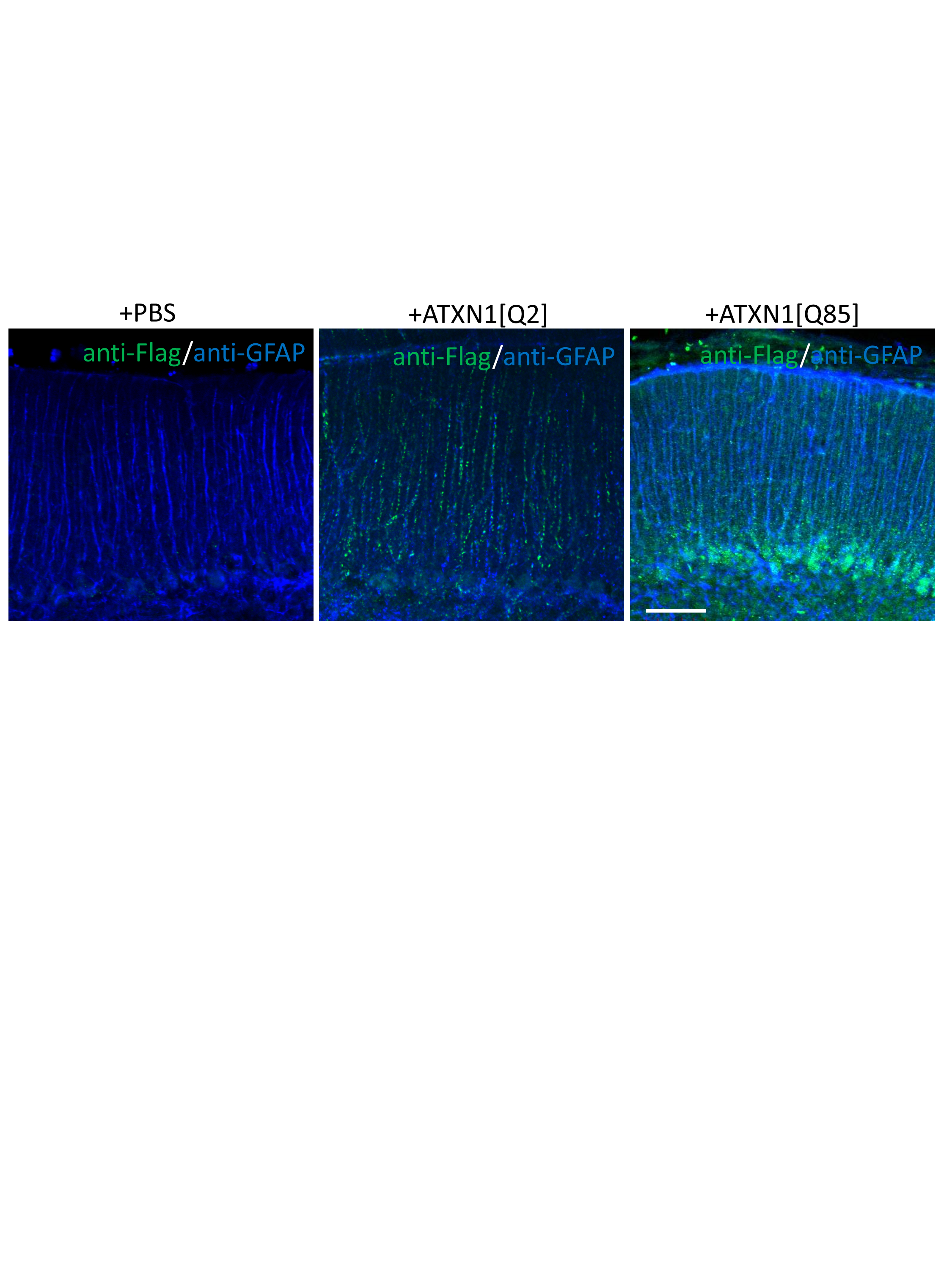

Supplement: Supplementary file 2 — Sup. Firure 1 [file 41420_2026_3120_MOESM2_ESM.tif]

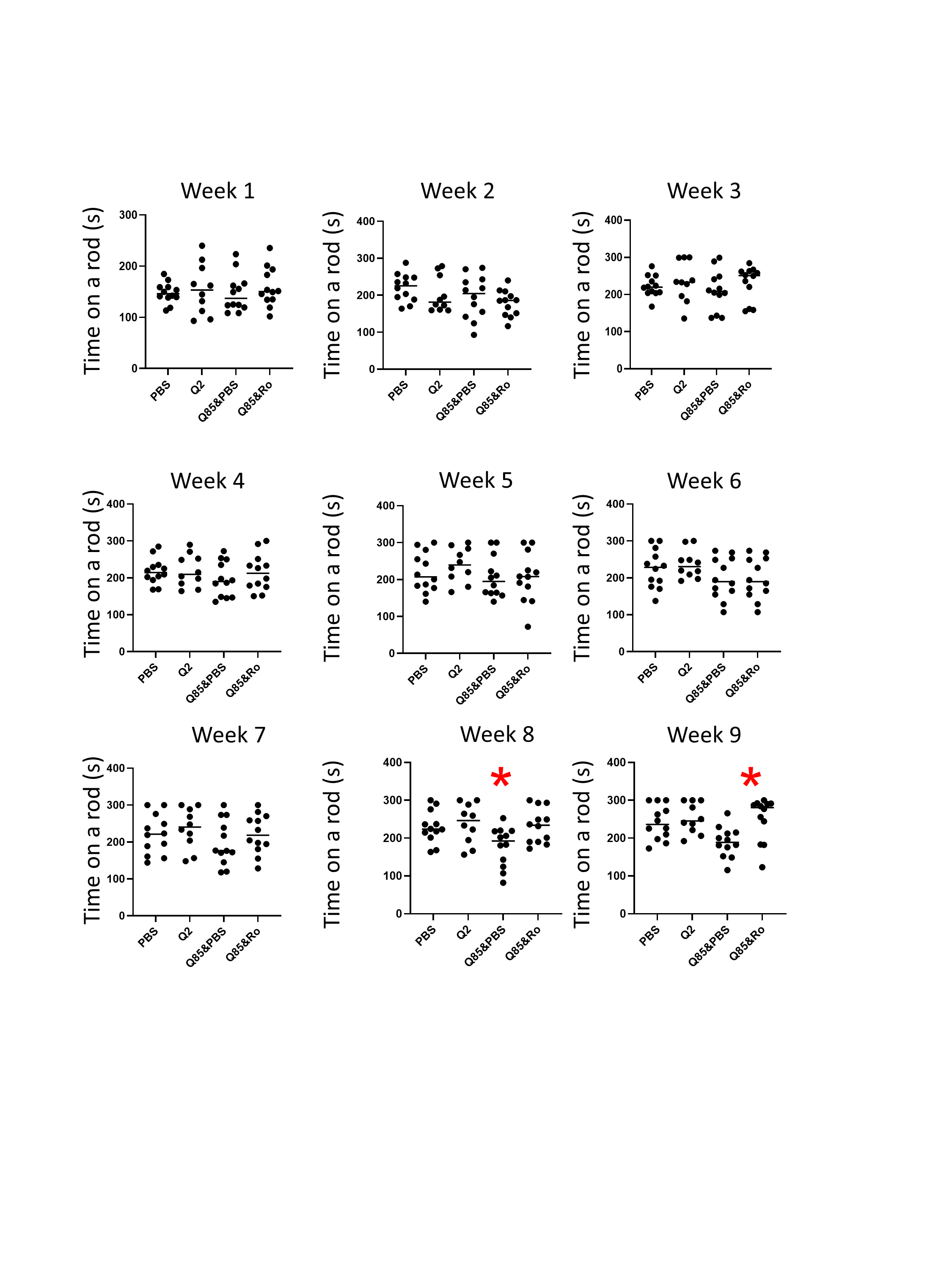

Supplement: Supplementary file 3 — Sup. Figure 2 [file 41420_2026_3120_MOESM3_ESM.tif]

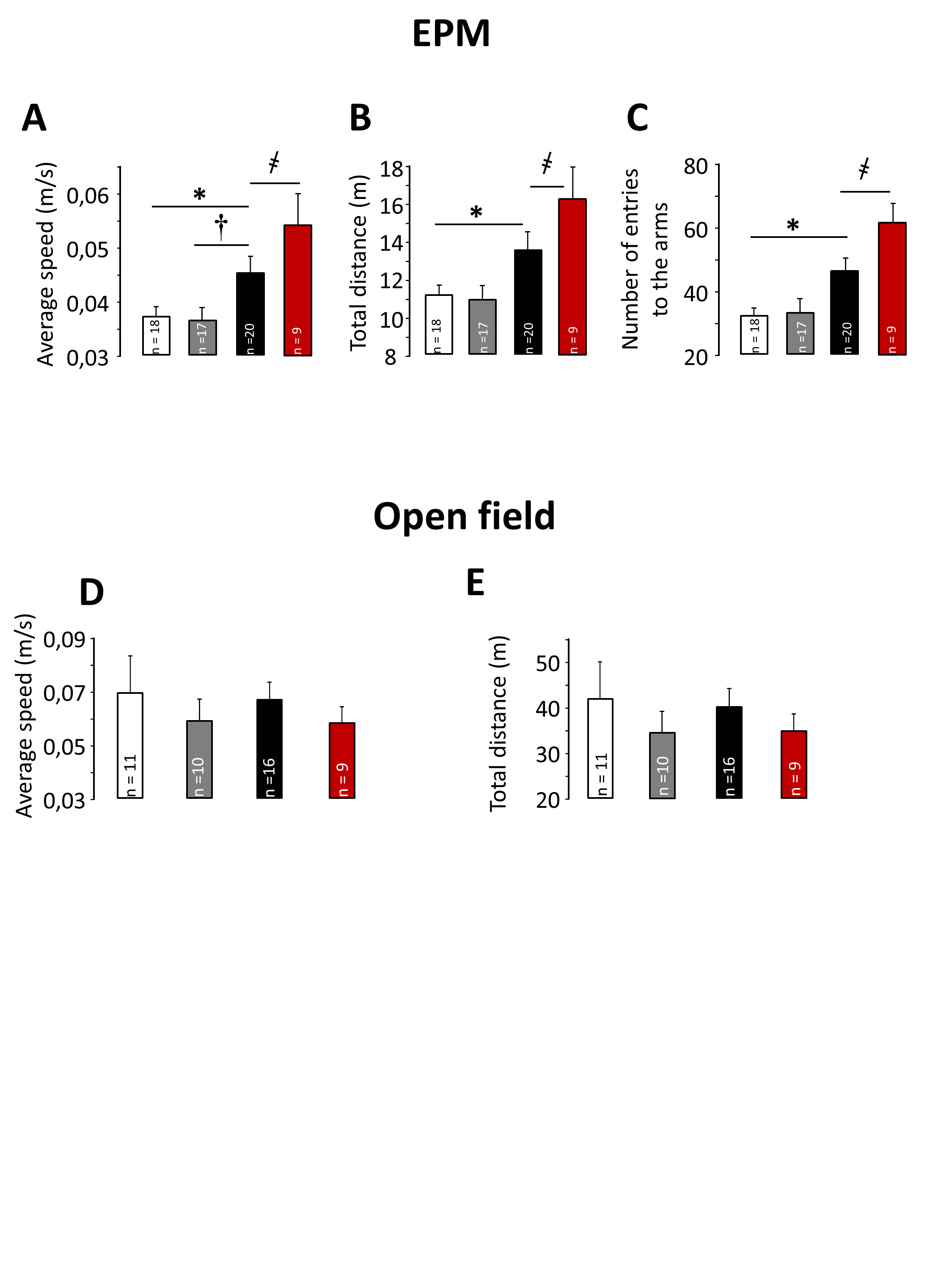

Supplement: Supplementary file 4 — Sup. Figure 3 [file 41420_2026_3120_MOESM4_ESM.tif]

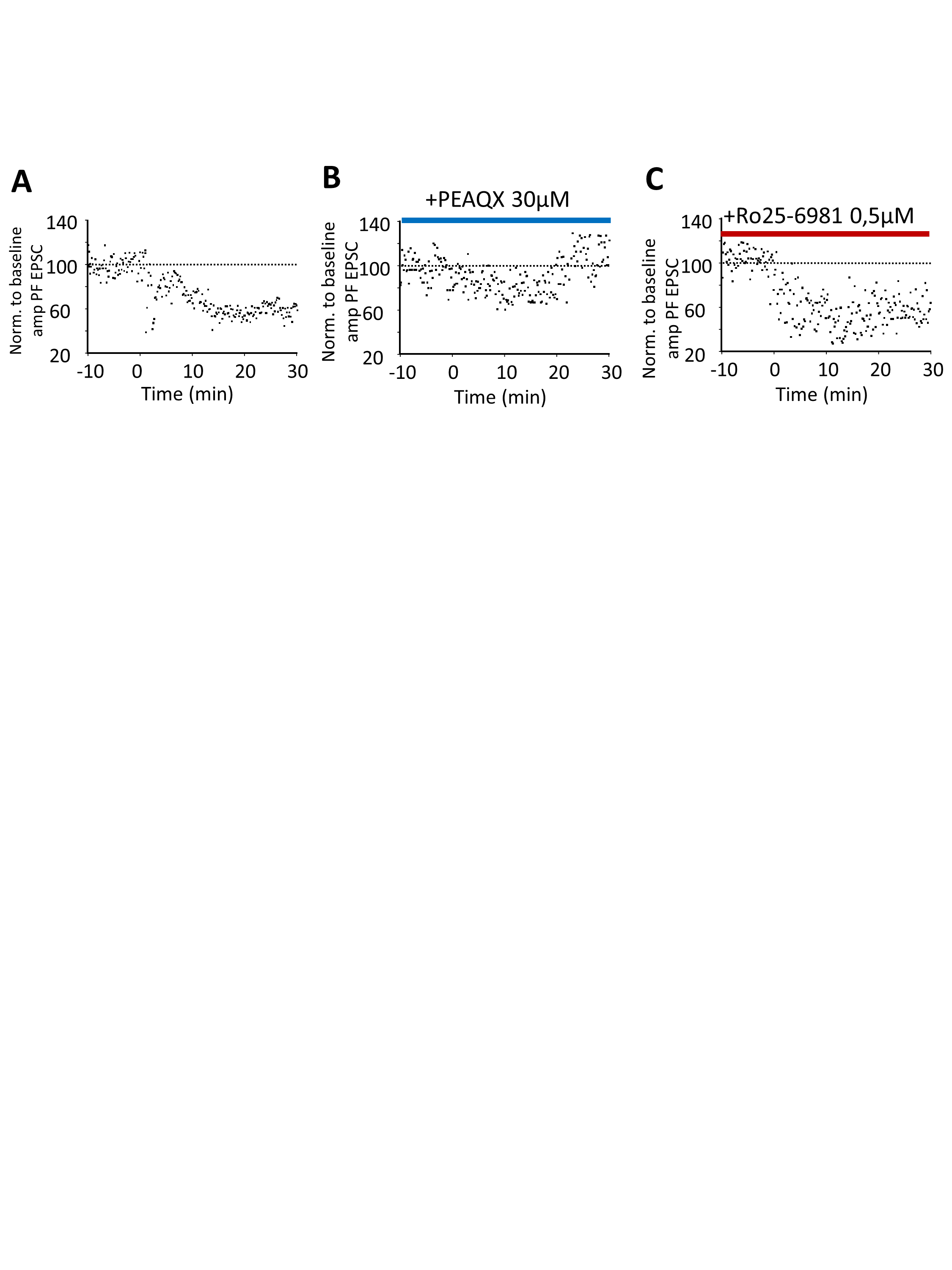

Supplement: Supplementary file 5 — Sup. Figure 4 [file 41420_2026_3120_MOESM5_ESM.tif]

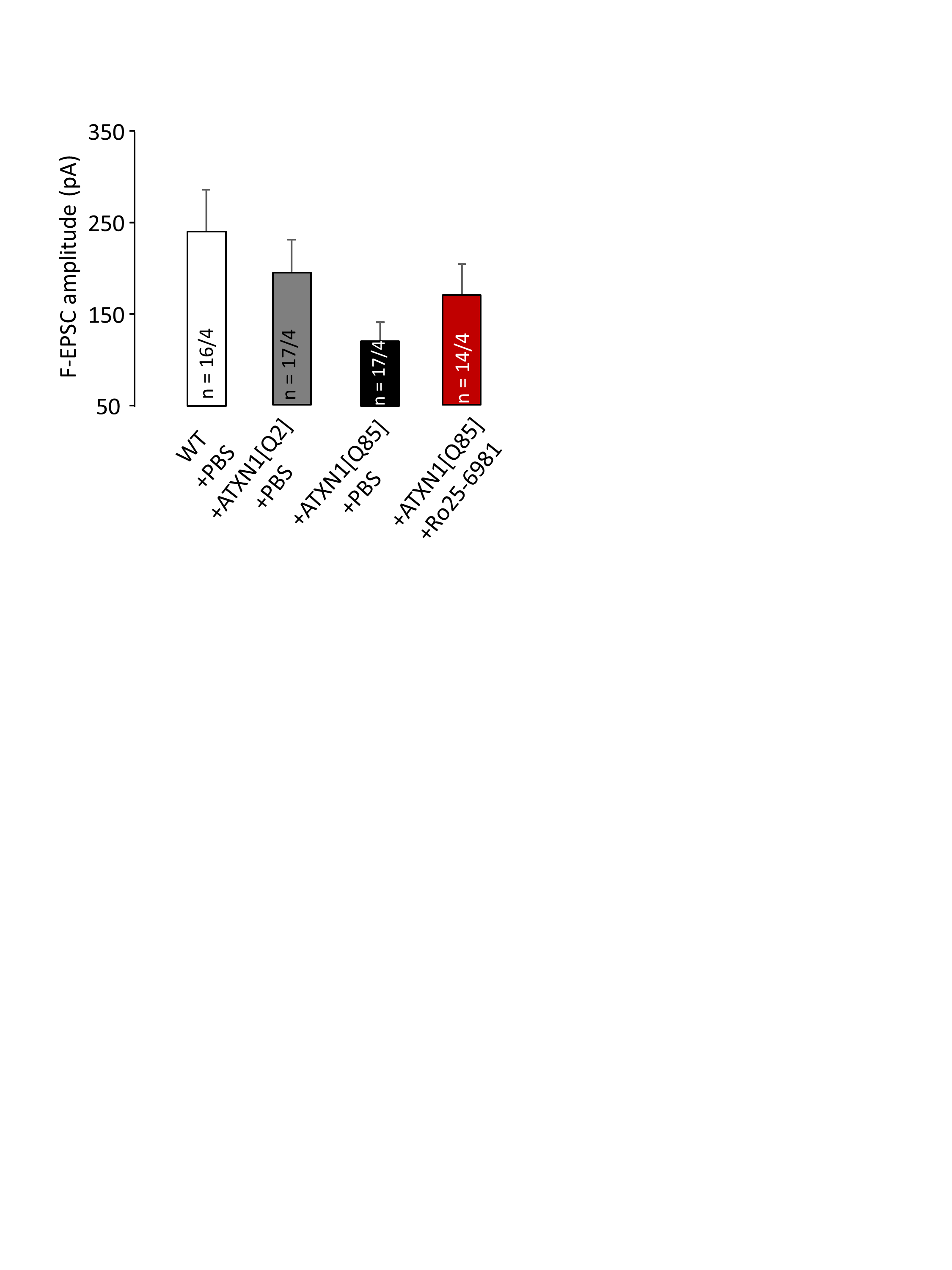

Supplement: Supplementary file 6 — Sup. Fig. 5 [file 41420_2026_3120_MOESM6_ESM.tif]

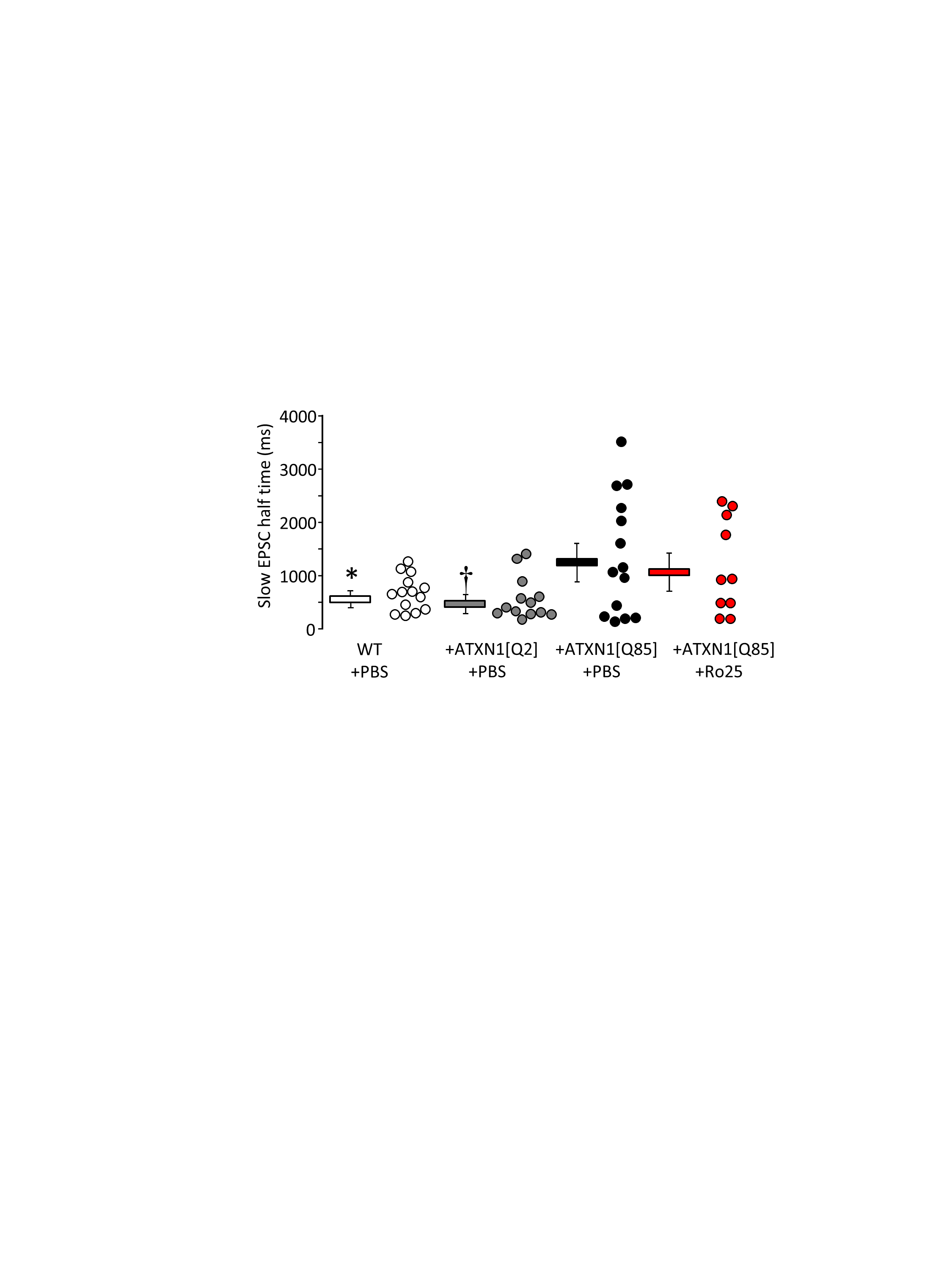

Supplement: Supplementary file 7 — Sup. Figure 6 [file 41420_2026_3120_MOESM7_ESM.tif]

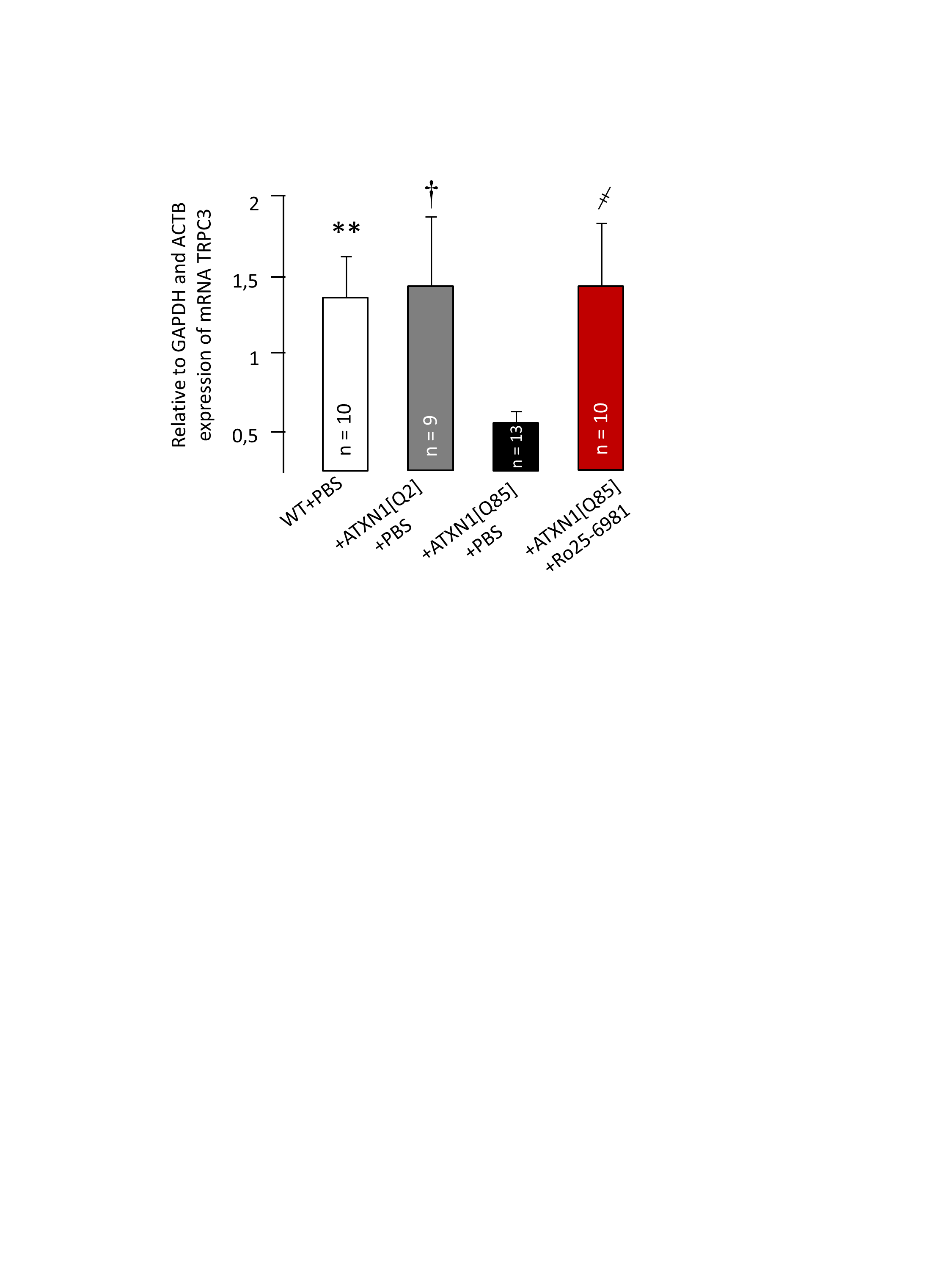

Supplement: Supplementary file 8 — Sup. Figure 7 [file 41420_2026_3120_MOESM8_ESM.tif]

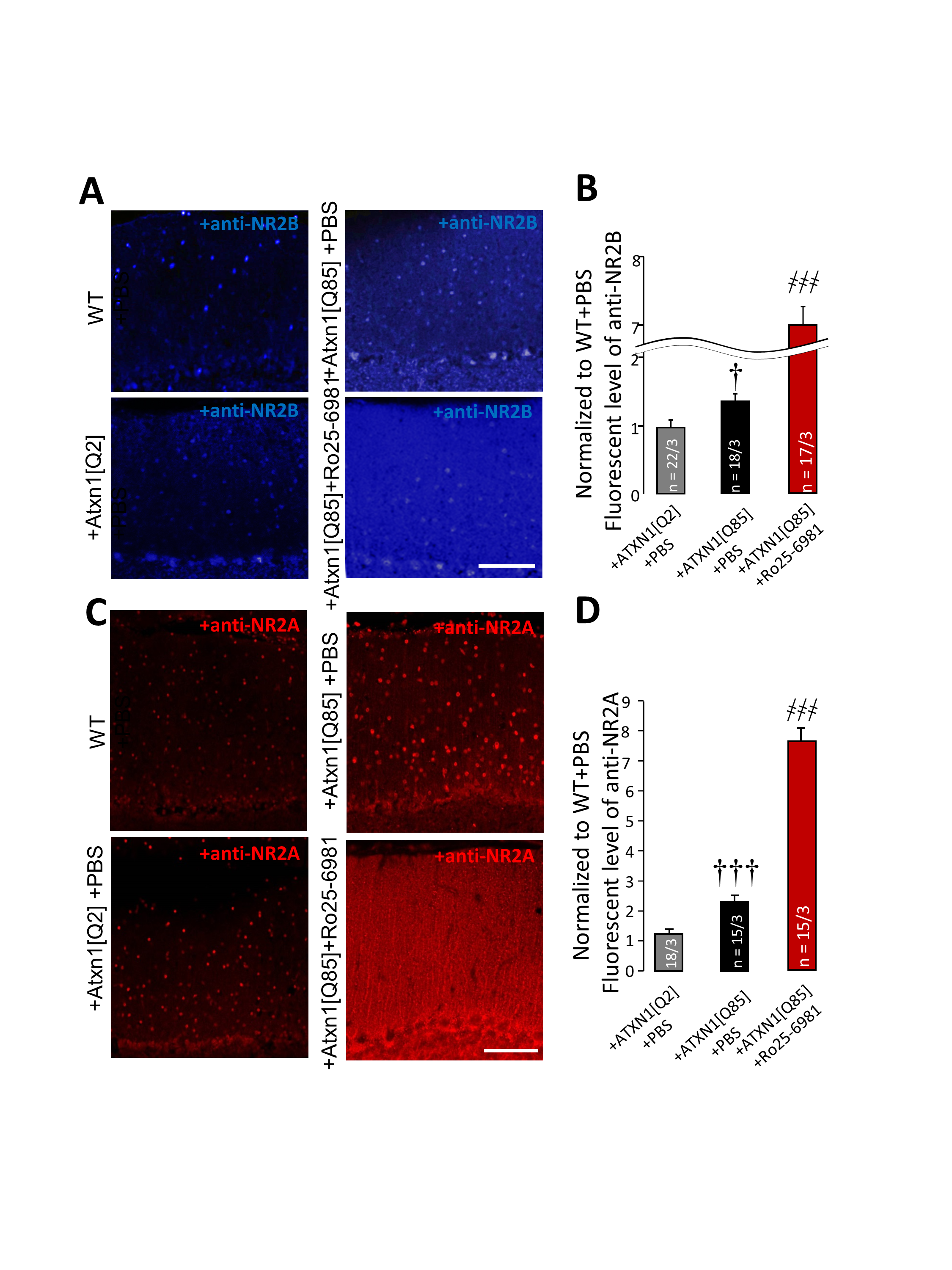

Supplement: Supplementary file 9 — Sup. Figure 8 [file 41420_2026_3120_MOESM9_ESM.tif]
