# Supplementary material for: Long term administration of selective NMDA GluN2B receptor blocker Ro25-6981 attenuates neurodegeneration in mouse model of spinocerebellar ataxia type 1 (SCA1)
Source: Cell Death Discov. 2026 Apr 13;12:228. doi: 10.1038/s41420-026-03120-z (PMC13184322; doi:10.1038/s41420-026-03120-z)
Supplement: Supplementary file 10 — Editable supplementary tables [file 41420_2026_3120_MOESM10_ESM.docx]

**Sup. Table 1**

|  | **Amp. EPSC (pA)** | **Rise time (ms)** | **τ**  **(ms)** |
| --- | --- | --- | --- |
| **Untreated (n = 10)** | 135.9±20.0 | 2.1±0.3 | 13.3±2.3 |
| **+Ro25-6981 (n = 10)** | 116.3±16.7 | 2.2±0.3 | 15.8±2.5 |
| **+MK801 (n = 10)** | 77.1±9.0 ^*^ | 2.0±0.4 | 15.6±2.7 |
| **+PEAQX (n = 10)** | 72.5±13.3 ^†^ | 2.2±0.5 | 17.9±2.4 |

**Sup. Table 2**

|  | Amp,  (pA) | Rise time (ms) | τ (ms) |
| --- | --- | --- | --- |
| **PBS (n=16/4)** | 239,2±46,3 | 2,8±0,2 | 16,8±1,6 |
| **Q2 (n=17/4)** | 196,3±34,6 | 2,6±0,2 | 15,3±1,3 |
| **Q85 + PBS (n=17/4)** | 120,0±21,0 | 1,9±0,2 | 17,0±2,5 |
| **Q85+Ro25 (n=14/4)** | 170,0±33,8 | 2,2±0,2 | 17,1±1,8 |
